# Two-carbon tethered artemisinin–isatin hybrids: design, synthesis, anti-breast cancer potential, and *in silico* study

**DOI:** 10.3389/fmolb.2023.1293763

**Published:** 2023-10-19

**Authors:** Ruo Wang, Renhong Huang, Yaofeng Yuan, Zheng Wang, Kunwei Shen

**Affiliations:** ^1^ Department of General Surgery, Comprehensive Breast Health Center, Ruijin Hospital, Shanghai Jiao Tong University School of Medicine, Shanghai, China; ^2^ Laboratory of Molecule Synthesis and Function Discovery (Fujian Province University), Department of Chemistry, Fuzhou University, Fuzhou, China

**Keywords:** breast cancer, artemisinin, isatin, hybrid molecules, drug resistance, *in silico* study

## Abstract

Eleven two-carbon tethered artemisinin–isatin hybrids (**4a–k**) were designed, synthesized, and evaluated for their antiproliferative activity against MCF-7, MDA-MB-231, and MDA-MB-231/ADR breast cancer cell lines, as well as cytotoxicity toward MCF-10A cells in this paper. Among them, the representative hybrid **4a** (IC_50_: 2.49–12.6 *µ*M) was superior to artemisinin (IC_50_: 72.4->100 *µ*M), dihydroartemisinin (IC_50_: 69.6–89.8 *µ*M), and Adriamycin (IC_50_: 4.46–>100 *µ*M) against the three tested breast cancer cell lines. The structure–activity relationship revealed that the length of the alkyl linker between artemisinin and isatin was critical for the activity, so further structural modification could focus on evaluation of the linker. The *in silico* studies were used to investigate the mechanism of the most promising hybrid **4a**. Target prediction, bioinformatics, molecular docking, and molecular dynamics revealed that the most promising hybrid **4a** may exert anti-breast cancer activity by acting on multiple targets such as *EGFR*, *PIK3CA*, and *MAPK8* and thus participating in multiple tumor-related signaling pathways.

## 1 Introduction

Breast cancer consists of a group of biologically and molecularly heterogeneous diseases that originate from the breast and can be divided into four major subtypes: luminal-A, luminal-B, human epidermal growth factor receptor 2 (HER2) positive, and triple-negative breast cancer (Feng et al., 2018; Wang et al., 2020a; Tsang and Tse, 2020). At present, breast cancer is the most prevalent malignancy in women (Akr et al., 2017; Lau et al., 2022). In 2020, breast cancer has overtaken lung cancer as the most common malignancy, and an estimated 2.3 million new cases of breast cancer were diagnosed, accounting for nearly one-fourth of women cancer patients (Jaiswal et al., 2021; Sung et al., 2021). Moreover, 685,000 women died due to breast cancer, leading to one-sixth of cancer-related deaths in women (Arnold et al., 2022). Unfortunately, the burden of breast cancer is expected to increase further in the coming years (Wang et al., 2020b; Schröder et al., 2022; Sher et al., 2022).

Advancements in the treatment of breast cancer have resulted in an increasing population of patients living with this disease, and chemotherapy occupies an important position in breast cancer therapy. However, the chemotherapy treatment suffers from the generation of drug resistance (Shaikh and Emens, 2022; Tufail et al., 2022). Hence, development of innovative chemotherapeutics is a promising strategy to improve therapeutic outcomes of breast cancer.

Hybrid molecules, generated by combining two or more molecule entities, could affect multiple targets to fight against various diseases including breast cancers (Bérubé, 2016; Waseem and Ahmad, 2022; Wang et al., 2023a). Artemisinin (ART) and its derivatives (dihydroartemisinin/DHA, artesunate, and artemether), from the traditional Chinese medicine drug, possess a unique peroxy bridge structure, and they could exert the anticancer effects through diverse mechanisms, inclusive of cell cycle inhibition, inhibition of tumor angiogenesis, promotion of DNA damage, and promotion of ferroptosis (Zhang S. et al., 2022a; Hu et al., 2022). Hence, ART derivatives may have significant therapeutic effects on cancers (Gao et al., 2020; Mancuso et al., 2021). Isatin is an exceptionally useful template for developing new anticancer scaffolds; on account of this finding, more and more isatin-based molecules are either in clinical use or in trials (Gupta et al., 2019; Ding et al., 2020). Accordingly, a combination of ART with isatin is a promising strategy to discover novel anti-breast cancer candidates.

In recent years, several series of ART–isatin hybrids have been screened for their potential against various cancer cell lines, and some of them demonstrated promising antiproliferative activity against breast cancer cells (Hou et al., 2021; Zhang Z. et al., 2022b; Dong et al., 2022; Wang et al., 2022). The structure–activity relationship (SAR) revealed that the linker between ART and isatin moieties influenced the antiproliferative activity significantly, and as a linker, alkyl was more favorable than 1,2,3-triazole. Hence, a series of two-carbon tethered ART–isatin hybrids were designed, synthesized, and assessed for their antiproliferative activity against both drug-sensitive (MCF-7 and MDA-MB-231) and Adriamycin-resistant (MDA-MB-231/ADR) breast cancer cell lines in this study. Moreover, the cytotoxicity of the synthesized ART–isatin hybrids toward normal MCF-10A breast cells was also tested. Finally, possible mechanisms were investigated by *in silico* studies. The purpose of this paper was to find the candidates with promising antiproliferative potential against both drug-sensitive and drug-resistant breast cancer cell lines and high selectivity.

## 2 Results and discussion

### 2.1 Experimental studies

#### 2.1.1 Synthesis

**SCHEME 1:**
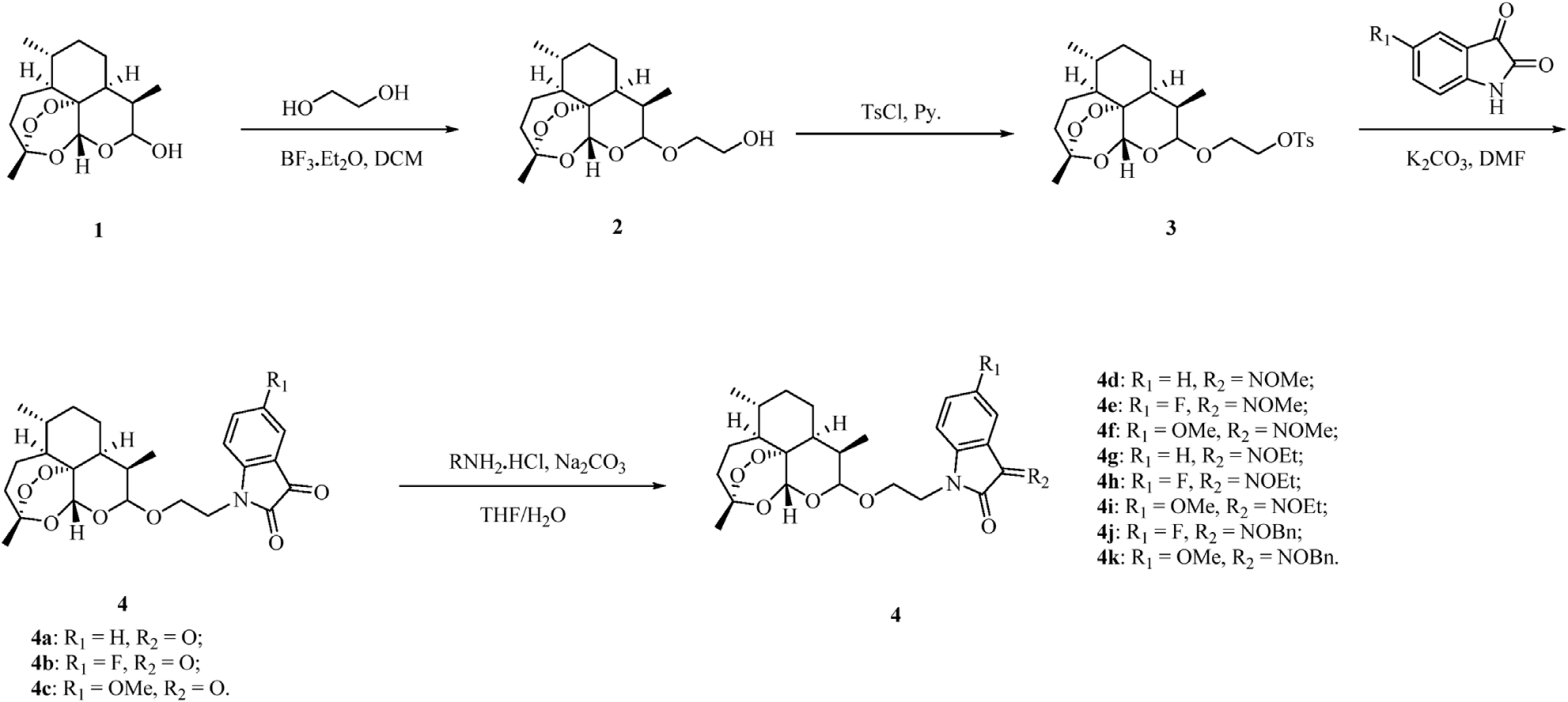
Synthetic route of two-carbon tethered ART–isatin hybrids **4a–k**.

The ART–isatin hybrids **4a–k** were prepared according to our method reported previously, and the detailed synthetic route is shown in Scheme 1 (Wang et al., 2023b). Etherification of dihydroartemisinin **1** with ethylene glycol in the presence of boron trifluoride diethyl etherate (BF_3_•OEt_2_) generated 2-hydroxyethyl dihydroartemisinin intermediate **2**. The conversion of the hydroxy group in intermediate **2** to tosylate (OTs) with pyridine as the base yielded intermediate **3**. Alkylation of (5-substituted)isatins with tosylate **3** provided desired ART–isatin hybrids **4a–c**. Finally, ART–isatin hybrids **4a–c** reacted with methoxylamine/ethoxylamine/benzyloxyamine hydrochlorides using sodium carbonate (Na_2_CO_3_) as the base yielded hybrids **4d–k**. The structures and yields are listed in Table 1.

**TABLE 1:** Structures and yields of two-carbon tethered ART–isatin hybrids **4a–k**.

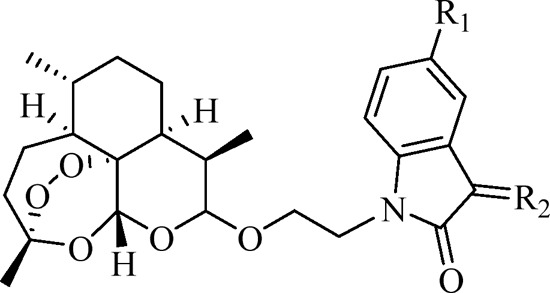			
Hybrid	R_1_	R_2_	Yield (%)
**4a**	O	H	53
**4b**	O	F	48
**4c**	O	OMe	51
**4d**	NOMe	H	86
**4e**	NOMe	F	94
**4f**	NOMe	OMe	81
**4g**	NOEt	H	65
**4h**	NOEt	F	88
**4i**	NOEt	OMe	80
**4j**	NOBn	F	69
**4k**	NOBn	OMe	67

#### 2.1.2 Characterization

The desired two-carbon tethered ART–isatin hybrids **4a–k** were characterized by high-resolution mass spectrometry (HRMS), proton nuclear magnetic resonance (^1^H NMR), and carbon-13 nuclear magnetic resonance spectroscopy (^13^C NMR) (Wang et al., 2023b). The corresponding analytical data and the analytical spectra are included in our previous study and Supplementary Figures S1–S33 **[**27**]**.

#### 2.1.3 Anti-breast cancer potential

The antiproliferative activity and cytotoxicity of **ART**–isatin hybrids **4a–k** against MCF-7 (CL-0149, purchased from Procell), MDA-MB-231 (CL-0150A, purchased from Procell), and MDA-MB-231/ADR (AC337895, purchased from CASMART) breast cancer cell lines, as well as MCF-10A (CL-0525, purchased from Procell), were assessed by the 3-(4,5-dimethylthiazol-2-yl)-2,5-diphenyltetrazolium bromide (MTT) assay. ART, DHA, and Adriamycin (ADR) were used as positive controls. The half-maximal inhibitory concentration (IC_50_) values are presented in Table 2. The selectivity index (SI: IC_50(MCF-10A)_/IC_50(MCF-7)_) and resistance index (RI: IC_50(MDA-MB-231/ADR)_/IC_50(MDA-MB-231)_) are presented in Table 3.

**TABLE 2:** Antiproliferative activity and cytotoxicity of ART–isatin hybrids **4a–k**.

Hybrid	Antiproliferative activity (IC_50_: *µ*M)^d^			Cytotoxicity (IC_50_: *µ*M)
	MCF-7	MDA-MB-231	MDA-MB-231/ADR^e^	MCF-10A
**4a**	12.6 ± 2.1	3.83 ± 0.1	2.49 ± 0.1	>100
**4b**	26.7 ± 2.4	18.6 ± 1.5	21.4 ± 3.1	>100
**4c**	33.8 ± 3.2	27.0 ± 2.2	25.1 ± 1.9	>100
**4d**	28.5 ± 2.3	16.2 ± 1.8	19.7 ± 1.2	>100
**4e**	73.3 ± 5.6	49.6 ± 3.8	64.7 ± 4.9	>100
**4f**	37.9 ± 3.3	20.4 ± 1.7	33.8 ± 3.0	>100
**4g**	48.2 ± 3.7	37.6 ± 2.4	28.4 ± 2.1	>100
**4h**	68.3 ± 5.2	46.5 ± 3.9	59.9 ± 4.4	>100
**4i**	70.8 ± 5.9	50.1 ± 4.2	52.2 ± 3.6	>100
**4j**	>100	>100	>100	>100
**4k**	>100	>100	>100	>100
**ART** ^a^	87.6 ± 6.6	72.4 ± 5.3	>100	>100
**DHA** ^b^	73.2 ± 5.9	69.6 ± 4.7	82.8 ± 6.8	>100
**ADR** ^c^	18.9 ± 1.2	4.46 ± 0.1	>100	68.8

^a^ ART, artemisinin.

^b^ DHA, dihydroartemisinin.

^c^ ADR, Adriamycin.

^d^ Data are represented as the mean of three independent experiments ±standard deviation S.D. (n = 3), where *p* ≤ 0.05.

^e^ MDA-MB-231/ADR: Adriamycin-resistant MDA-MB-231 cell line.

**TABLE 3:** Selectivity index and resistance index of ART–isatin hybrids **4a–k**.

Hybrid	SI^a^	RI^b^
**4a**	>7.9	0.65
**4b**	>3.7	1.15
**4c**	>2.9	0.93
**4d**	>3.5	1.22
**4e**	>1.3	1.30
**4f**	>2.6	1.66
**4g**	>2.0	0.75
**4h**	>1.4	1.28
**4i**	>1.4	1.04
**4j**	-	-
**4k**	-	-
**ART** ^c^	>1.1	>1.38
**DHA** ^d^	>1.3	1.18
**ADR** ^e^	3.6	22.4

^a^ SI, selectivity index, IC_50(MCF-10A)_/IC_50(MCF-7)_.

^b^ RI, resistance index, IC_50(MDA-MB-231/ADR)_/IC_50(MDA-MB-231)_.

^c^ ART, artemisinin.

^d^ DHA, dihydroartemisinin.

^e^ ADR, Adriamycin.

Table 2 demonstrates that a significant part of the synthesized hybrids (IC_50_: 2.49–73.3 *µ*M) showed considerable activity against MCF-7, MDA-MB-231, and MDA-MB-231/ADR breast cancer cell lines, and the activity was superior to that of ART (IC_50_: 72.4->100 *µ*M) and DHA (IC_50_: 69.6–89.8 *µ*M). The SAR illustrated that the introduction of the (methoxy/ethoxy/benzyloxy)imino group into the C-3 position and fluoro or methoxy group into the C-5 position of the isatin moiety decreased the activity. In particular, incorporation of the benzyloxyimino group into the C-3 position of isatin skeleton led to a loss of activity. Moreover, the length of the alkyl linker between ART and isatin seems to have a significant influence on the activity, as evidenced by that the two-carbon tethered ART–isatin hybrids were more potent than the three-carbon analogs reported in reference 17.

All the synthesized two-carbon tethered hybrids (IC_50_: >100 *µ*M) were non-toxic toward normal MCF-10A breast cancer cells as ART (IC_50_: >100 *µ*M) and DHA (IC_50_: >100 *µ*M), and the cytotoxicity was lower than that of Adriamycin (IC_50_: 68.8 *µ*M).

Table 3 shows that the selectivity index (SI: IC_50(MCF-10A)_/IC_50(MCF-7)_) values of six hybrids were >2.0, which were similar to the previously reported ART–isatin hybrids and controls ART (SI: >1.1), DHA (SI: >1.3), and ADR (SI: 3.6) (Hou et al., 2021; Zhang Z. et al., 2022b; Dong et al., 2022; Wang et al., 2022). It was worth noting that the resistance index (RI: IC_50(MDA-MB-231/ADR)_/IC_50(MDA-MB-231)_) values of the active hybrids **4a–k** were in a range of 0.65–1.66, which were comparable to the previously reported ART–isatin hybrids and controls ART (RI: >1.38) and DHA (RI: 1.18), and lower than ADR (RI: 22.4), indicating that these hybrids had no or low cross resistance with the current available anti-breast cancer agents (Hou et al., 2021; Zhang Z. et al., 2022b; Dong et al., 2022; Wang et al., 2022).

Amongst the synthesized hybrids, the most active hybrid **4a** (IC_50_: 2.49–12.6 *µ*M) not only was far more potent than the parents ART (IC_50_: 72.4->100 *µ*M) and DHA (IC_50_: 69.6–89.8 *µ*M) but also possessed higher activity than Adriamycin (IC_50_: 4.46->100 *µ*M) against all the three breast cancer lines. The RI value of hybrid **4a** was 0.65, implying its potential to overcome drug resistance. In addition, hybrid **4a**: >100 *µ*M) was non-toxic toward normal MCF-10A breast cells, and the SI value was >7.9. Accordingly, hybrid **4a** could serve as a promising candidate for further preclinical evaluations.

### 2.2 *In silico* studies

#### 2.2.1 Target prediction of 4a

SwissTargetPrediction, a tool for predicting the targets of compounds based on the similarity of two-dimensional and three-dimensional structures of known compounds, has become a common tool for predicting the targets of compounds currently due to its better sensitivity and specificity (Daina et al., 2019). We selected the promising candidate **4a** and performed target prediction by SwissTargetPrediction. The results showed that **4a** may have 109 potential targets. Supplementary Table S1 displays the full list in detail.

#### 2.2.2 Construction of cross-over genes, protein–protein interaction networks, and enrichment analysis

**FIGURE 1:**
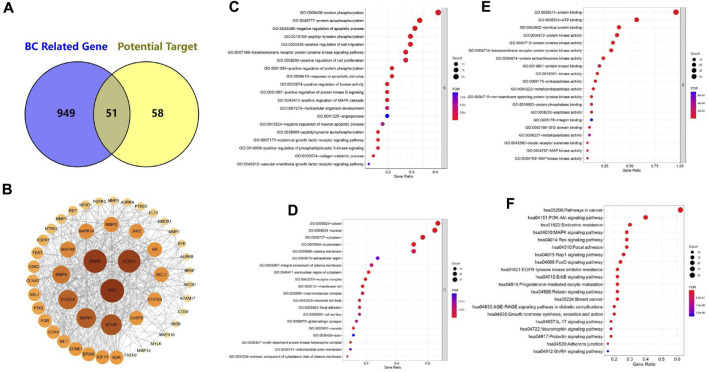
**(A)** Intersection of BC-related gene and potential target (Venn diagram). **(B)** Protein–protein interaction (PPI) network diagram of candidate targets. **(C–E)** GO enrichment analysis of target genes: **(C)** biological processes, **(D)** cellular components, and **(E)** molecular functions. **(F)** KEGG enrichment analysis of target genes.

To better explore the cross-over genes, we selected breast cancer-related gene (BC-related gene) in GeneCard to intersect with our predicted targets of **4a** (potential target) (Safran et al., 2010). Finally, a total of 51 cross-over genes were selected (Figure 1A). These cross-over genes are the potential gene targets of **4a** in the activities against breast cancer. Supplementary Table S2 displays the full list in detail.

To obtain the protein–protein interaction (PPI) networks, we imported 51 cross-over genes into the STRING database and selected *Homo sapiens* as the organism. The results showed 462 interactions. Subsequently, the PPI networks were obtained using Cytoscape for visualization and calculation (Figure 1B). The average closeness centrality (CC) was 0.62, the average betweenness centrality (BC) was 31.5, and the average degree value (DV) was 18.5. Among them, *SRC*, *EGFR*, *CCND1*, *MTOR*, *MAPK1*, *PIK3CA*, *MMP9*, *MAPK8*, *MMP2*, and *MAPK14* had higher DV, BC, and CC values than the norm. This suggests that they are the central targets of **4a**. Supplementary Table S3 displays the specific node properties in detail.

We performed Gene Ontology (GO) enrichment analysis on selected target genes to further examine them. Based on the GO enrichment analysis, most of the target genes were identified to be involved in biological processes (BPs), cellular components (CCs), and molecular functions (MFs). BP enrichment was mainly involved in the following processes (Figure 1C): protein phosphorylation (21/51), protein autophosphorylation (17/51), negative regulation of the apoptotic process (16/51), and peptidyl-tyrosine phosphorylation (15/51). Positive CC enrichment was observed in the following compartments (Figure 1D): cytosol (34/51), nucleus (33/51), cytoplasm (29/51), nucleoplasm (26/51), and plasma membrane (26/51). MF enrichment includes (Figure 1E) protein binding (49/51), ATP binding (29/51), identical protein binding (20/51), and protein kinase activity (19/51).

We also performed Kyoto Encyclopedia of Genes and Genomes (KEGG) enrichment analysis on these target genes, and the KEGG pathway enrichment analysis showed that the target genes were mainly involved in pathways in cancer (31/51), the PI3K-Akt signaling pathway (20/51), endocrine resistance (15/51), MAPK signaling pathway (14/50), Ras signaling pathway (14/51), and other pathways (Figure 1F). These suggest that **4a** may exert anti-breast cancer effects by acting on multiple tumor signaling pathways.

#### 2.2.3 Molecular docking

Molecular docking can be used to assess **4a**-central target protein interactions. Generally, lower binding energy suggests better interactions. For now, there is no single criterion for screening the binding energy. The screening is usually based on a binding energy ≤ −5.0 kcal/mol (Zhang J. et al., 2022c). The results of molecular docking showed that 7 of the 10 selected central targets were below −5.0 kcal/mol. These results suggest that **4a** has good interactions with the seven central targets. All the results are shown in Table 4.

**TABLE 4:** **4a**-target molecular docking binding energy.

Target	PDB ID	Binding energy (kcal/mol)
EGFR	7SYD	−6.857
MAPK8	2XRW	−6.305
PIK3CA	7PG5	−6.082
MTOR	6ZWO	−5.818
MAPK14	3LFF	−5.733
MAPK1	8AOJ	−5.203
CCND1	2W96	−5.004
SRC	2H8H	−4.628
MMP2	1CK7	−4.577
MMP9	5UE3	−3.561

In addition, the visualization analysis allowed us to better understand the spatial structure and interactions between **4a** and the central targets. The intermolecular force between **4a** and the central targets include van der Waals, hydrogen bond (conventional hydrogen bond, etc.), and hydrophobic (pi–alkyl, alkyl, etc.) forces. Notably, **4a** also generates interesting intermolecular forces with central targets, which include pi–sulfur with EGFR, pi–anion with MAPK1 and MAPK14, pi–pi T-shaped with MMP9, pi–cation with MAPK8, amide–pi stacked with MAPK8, and pi–pi stacked with MMP2. These interesting interactions may have a specific effect on the affinity of the **4a**-central marker and deserve further investigation. All the results are shown in Supplementary Figures S34–S43.

#### 2.2.4 Molecular dynamics

In order to better understand the kinetics of the interaction between **4a** and target proteins, we selected three proteins with the best binding energy—EGFR, MAPK8, and PIK3CA—as key target proteins for molecular dynamics simulation studies.

**FIGURE 2:**
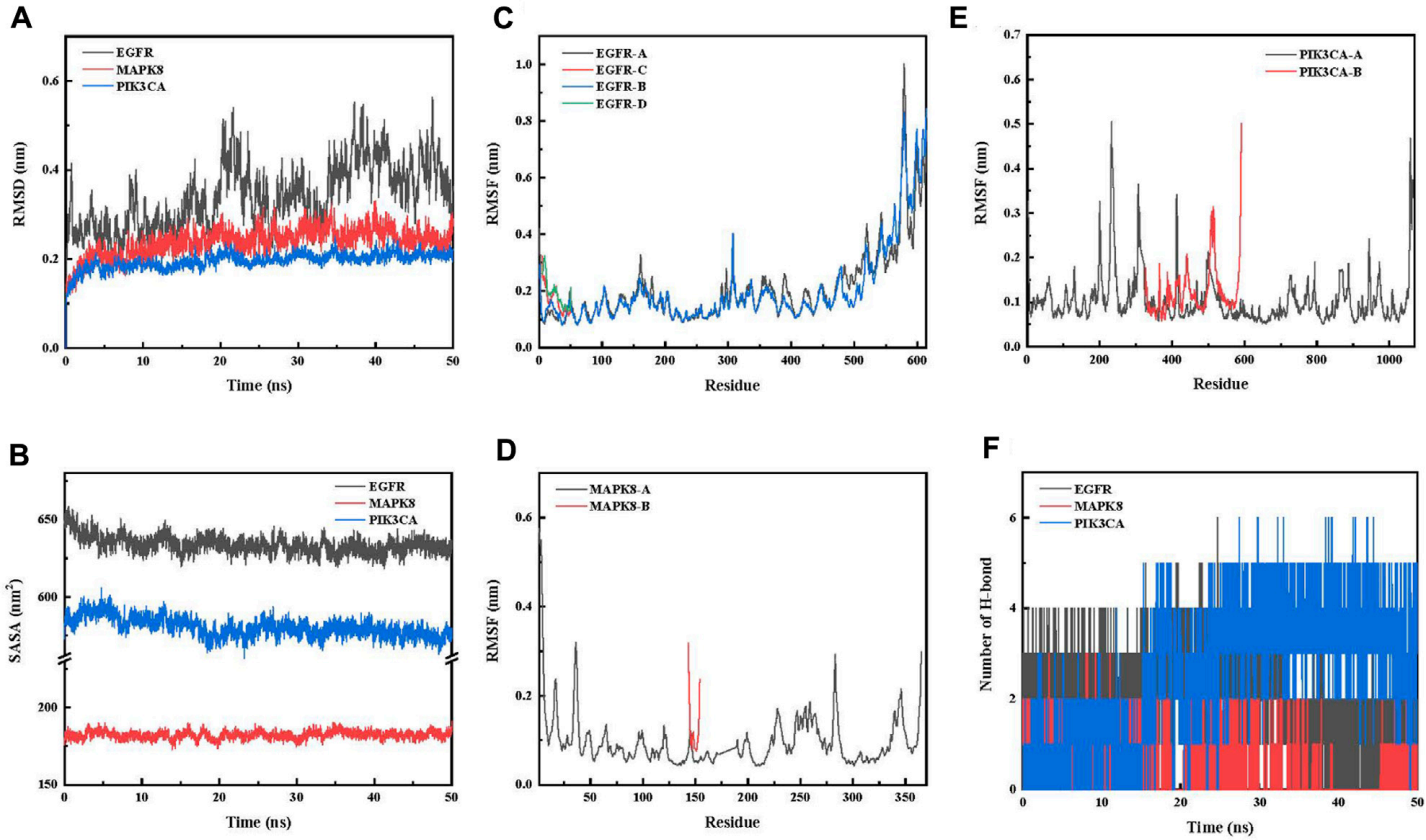
**(A)** Variation curve of RMSD of the protein with time during the simulation of the **4a**-key target protein. **(B)** Variation curve of SASA of the protein with time during the simulation of the **4a**-key target protein. **(C)** RMSF analysis of **4a**-EGFR. **(D)** RMSF analysis of **4a**-MAPK8. **(E)** RMSF analysis of **4a**-PIK3CA. **(F)** Variation in the number of hydrogen bonds with time during the simulation of the **4a**-key target protein.

Root-mean-squared deviation (RMSD), which measures the coordinate deviation of a specific atom with respect to a reference structure, is often used to assess whether a ligand–receptor system has reached stability (Sargsyan et al., 2017). A stable RMSD means that the atoms in the corresponding system become stable, whereas a fluctuating RMSD implies fluctuations. This simulation process is 50 ns (Figure 2A). The simulations show that **4a**-MAPK8 and **4a**-PIK3CA reach equilibrium soon after the start of the simulation process and that the RMSD values are below 0.3 nm throughout the simulation time. This suggests that the protein–ligand system fluctuates less, which indicates that the docking level is reasonable in the dynamics study (Rastelli et al., 2010; Dai et al., 2023). **4a**-EGFR fluctuated more in the initial stage and reached equilibrium after 32 ns, and the RMSD value was approximately 0.5 nm throughout the simulation time, suggesting poor stability in the dynamics study (Li et al., 2010).

The solvent-accessible surface area (SASA) is calculated by the van der Waals forces interacting with solvent molecules to calculate the solute area (Akoonjee et al., 2022). The lower SASA can be explained by stronger hydrophobic interactions and less inter-complex solvent water, that is, the more compact binding between the **4a**-key target protein complexes (VAN DAN BURG et al., 1994). The 50-ns simulation results (Figure 2B) show that all **4a**-key target proteins show an acceptable SASA value of the protein–ligand complex during the complex simulation. In addition, the results indicate that the **4a**-PIK3CA complex exhibits the most compact structure, followed by the **4a**-MAPK8 complex, while the **4a**-EGFR complex is the the loosest structure in the dynamics study (Zhou et al., 2023).

Root-mean-squared fluctuation (RMSF) calculates the increase and decrease of each atom relative to its average position, characterizing the change in structure averaged over time, i.e., giving a characterization of the flexibility of each region of the protein (Işık et al., 2022; Xu et al., 2022). All results are shown in Figures 2C–E. First, the residues with small RMSF fluctuations in the protein complex system are consistent with the active residues, which may be related to the interactions such as hydrogen bonds and hydrophobic interactions generated between **4a**-key target proteins and forming stable compounds. The regions with larger fluctuations are located in the inactive regions at the edges of the protein, which may be related to interactions such as water and chloride ions. The RMSF values of the **4a**-key target protein showed little fluctuation during the 50-ns simulation, indicating that the protein ligands can bind stably in the dynamics study (Radwan et al., 2022).

To explore the interaction of **4a** with key target proteins, first, we performed hydrogen bonding analysis (Figure 2F). The average numbers of hydrogen bonds of **4a**-EGFR, **4a**-MAPK8, and **4a**-PIK3CA were 1.97, 0.58, and 2.63, respectively, suggesting that the binding stability of **4a**-PIK3CA may be higher than that of the **4a**-EGFR and **4a**-MAPK8 systems in the dynamics study.

Molecular mechanics-Poisson–Boltzmann surface area (MM-PBSA) is a method for post-processing molecular dynamics trajectories to estimate binding free energies (Homeyer and Gohlke, 2012). To better explain the interaction energy between the ligand and the receptor, we determined the binding energy of all protein–ligand complexes in the equilibrium phase using the gmx_mmpbsa method (Valdés-Tresanco et al., 2021). In the application of the MM-PBSA method, the total binding energy was decomposed into four independent components (electrostatic interactions, van der Waals interactions, and polar and nonpolar solvation interactions). The results of the binding energy of **4a**-key target protein are shown in Supplementary Table S4. In the **4a**-key target protein complex system, the total binding energies were all negative, indicating their contribution to the binding of the complex system. Among them, the total binding free energies of **4a**-EGFR, **4a**-MAPK8, and **4a**-PIK3CA were −72.310 kJ/mol, −109.794 kJ/mol, and −110.833 kJ/mol, respectively. These results indicate that the total binding free energies of the **4a**-key target protein complex system supported the strong binding of **4a**-MAPK8 and **4a**-PIK3CA complexes, while the **4a**-EGFR complex is relatively weaker in the dynamic system (Fu et al., 2017; Kushwaha et al., 2021).

To further understand the **4a**-key target protein interaction, we assessed the interaction of each residue with **4a** by decomposing the total binding energy into the per-residue contribution energy (Supplementary Figures S44A–C). The important contributing amino acid residues of the **4a**-EGFR complex are mainly HIS209 (−8.794 kJ/mol), CYS207 (−5.581 kJ/mol), and ASN210 (−4.140 kJ/mol) in the B-chain of EGFR and HIS209 (−4.043 kJ/mol) in the A-chain of EGFR. The **4a**-MAPK8 complex mainly receives contributions from amino acid residues VAL40 (−9.077 kJ/mol), LEU168 (-5.324 kJ/mol), and VAL158 (−4.171 kJ/mol) in the A-chain of MAPK8. LYS678 (−6.364 kJ/mol) and THR679 (−4.450 kJ/mol) in the A-chain of PIK3CA and GLN375 (−4.731 kJ/mol) in the B-chain of PIK3CA are the major amino acid residues of PIK3CA interacting with **4a**.

## 3 Conclusion

**FIGURE 3:**
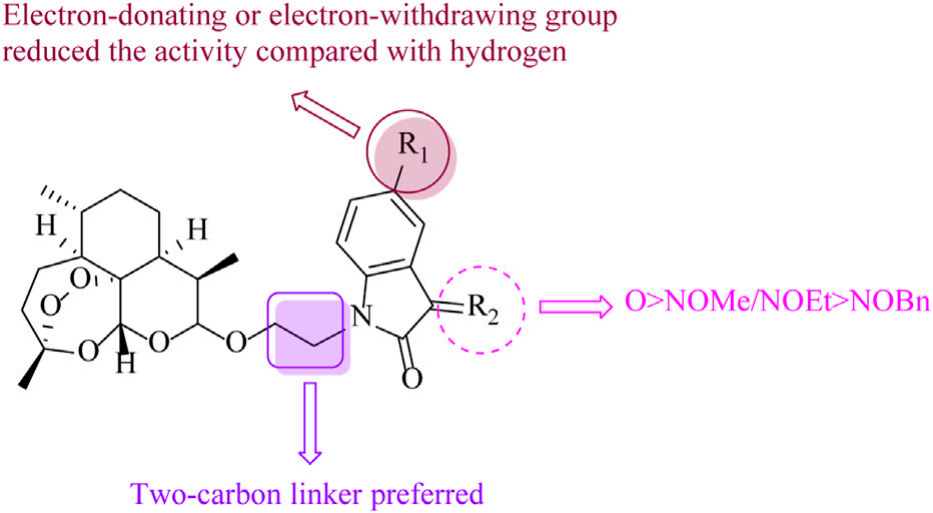
SAR of ART–isatin hybrids **4**.

In summary, a series of two-carbon tethered ART–isatin hybrids were synthesized and assessed for their antiproliferative activity against MCF-7, MDA-MB-231, and MDA-MB-231/ADR breast cancer cell lines, as well as cytotoxicity toward MCF-10A cells. SAR (Figure 3) revealed that 1) the length of the alkyl linker between ART and isatin influenced the activity remarkably, and the two-carbon linker was more favorable than the three-carbon linker; 2) the relative contribution order of the functional group at the C-3 position of isatin moiety was carbonyl > methoxy/ethoxyimino > benzyloxyimino; 3) compared with hydrogen, regardless of the electron-donating or electron-withdrawing group at the C-5 position of isatin skeleton, the activity was reduced.

In particular, the representative hybrid **4a** (IC_50_: 2.49–12.6 *µ*M) not only was superior to Adriamycin (IC_50_: 4.46->100 *µ*M) against all the three breast cancer lines but also exhibited an excellent safety profile and had the potential to overcome drug resistance. Meanwhile, the *in silico* study provided a preliminary mechanistic study of **4a**. The PPI network suggested that **4a** may target related proteins expressed by *SRC*, *EGFR*, *CCND1*, *MTOR*, *MAPK1*, *PIK3CA*, *MMP9*, *MAPK8*, *MMP2*, *MAPK14*, and other genes, which may be central targets for **4a** to exert its anti-breast cancer effects. KEGG enrichment analysis suggested that **4a** may exert anti-breast cancer effects by participating in the PI3K-Akt signaling pathway, endocrine resistance, MAPK signaling pathway, and other breast cancer-related pathways. Subsequently, molecular docking revealed the binding energy of **4a** to the central target and detailed intermolecular force information. Finally, molecular dynamics studies provided dynamic information on the binding pattern of **4a** to the three highest interacting key targets (EGFR, MAPK8, and PIK3CA). Therefore, hybrid **4a** was a promising anti-breast cancer candidate and merited further preclinical evaluations.

## Data Availability

The original contributions presented in the study are included in the article/Supplementary Materials; further inquiries can be directed to the corresponding authors.
