# Supplementary material for: Two-carbon tethered artemisinin–isatin hybrids: design, synthesis, anti-breast cancer potential, and in silico study
Source: Front Mol Biosci. 2023 Oct 19;10:1293763. doi: 10.3389/fmolb.2023.1293763 (PMC10620963; doi:10.3389/fmolb.2023.1293763)
Supplement: Supplementary file 1 [file DataSheet1.docx]

**Supplementary Materials**

**Two-carbon tethered artemisinin-isatin hybrids: Design, Synthesis, anti-breast cancer potential and in silico study**

**Experimental section**

**Materials**

^1^H NMR and ^13^C NMR spectra were determined on a Varian Mercury-400 spectrometer in CDCl_3_ using tetramethylsilane (TMS) as an internal standard. Electrospray ionization (ESI) mass spectra were obtained on a MDS-SCIEX-Q-Tap Mass Spectrometer with our previous study [1]. Unless otherwise noted, the reagents were obtained from commercial supplier and were used without further purification. MCF-7 (CL-0149), MDA-MB-231 (CL-0150A) breast cancer cell lines were purchased from Procell. MCF-10A (CL-0525) normal breasr cell was purchased from Procell. MDA-MB-231/ADR (AC337895) adriamycin-resistant breast cancer cell lines were purchased from Casmart.

**Synthesis**

To the mixture of dihydroartemisinin **1** (100 mmol) and ethylene glycol (120 mmol) in Dichloromethane (300 mL) was added Boron trifluoride-diethyl etherate (30 mL) at 0 ^o^C. The mixture was stirred at 25 ^o^C overnight, and then sat. Na_2_CO_3_ (500 mL) was added dropwise to quench the reaction. The organic layer was washed with brine (200 mL), dried over anhydrous Na_2_SO_4_, filtrated, and concentrated under reduced pressure. The residue was purified by silica gel chromatography eluted with PE to PE: EA = 1:1 to give 2-hydroxyethyl dihydroartemisinin **2**. To the solution of 2-hydroxyethyl dihydroartemisinin **2** (50 mmol) in pyridine (250 mL), *p*-toluenesulfonyl chloride (60 mmol) in was added at 0 ^o^C, and the mixture was stirred at room temperature for 5 h. The mixture was concentrated *in vacuo* to give crude toslyate **3** which was used directly in the next step. The suspension of isatin/5-fluoroisatin/5-methoxyisatin (15 mmol), crude toslyate **3** (10 mmol) and potassium carbonate (K_2_CO_3_, 20 mmol) in DMF (60 mL) was stirred overnight, and then filtered. The filtrate was concentrated under reduced pressure to give the residue which was purified by silica gel chromatography eluted with PE to PE:EA = 1:1 to give desired ART-isatin hybrids **4a-c**. To a solution of hybrids **4a-c** (1 mmol) and methoxylamine/ethoxylamine/benzyloxyamine hydrochlorides (2 mmol) in a mixture of THF (10 mL) and H_2_O (10 mL), sodium carbonate (Na_2_CO_3_, 3.0 mmol) was added. The mixture was stirred at 60 ^o^C for 12 h, and the cooled to room temperature. The mixture was extracted with EA (30 mL×3), and the combined organic layers were washed with H_2_O (30 mL), dried over anhydrous Na_2_SO_4_, filtrated, and concentrated under reduced pressure. The residue was purified by silica gel chromatography eluted with PE to PE: EA = 1:1 to give hybrids **4d-k**.

***In vitro* antiproliferative activity evaluation**

MCF-7, MDA-MB-231 and MDA-MB-231/ADR breast cancer cells (2×10^3^) were plated in each well of a 96-well plate and were allowed to adhere and spread for 24 h. Cells were treated with a range of different concentrations of ART-isatin hybrids **4a-k** (1, 5, 10, 20, 50, 100 *µ*M) and reference drugs artemisinin, dihydroartemisinin, and adriamycin (1, 5, 10, 20, 50, 100 *µ*M) and were cultured for 24 h at 37 ^o^C. 3-(4,5-dimethyl-2-thiazolyl)-2,5-diphenyltetrazolium bromide (MTT) solution (10 *µ*L) was added to each well, and the cultures were incubated for an additional 4 h. A further 100 *µ*L of MTT solution was added and incubation continued overnight. The absorbance was read at 540 nm in EnSpire, multimode reader and IC_50_ concentration was calculated and indicated as means SD of three in-dependent experiments. The percentage relative cell viability was determined according to the following formula: Percentage cell viability = A at 570 nm of treated samples/A at 570 nm of untreated samples × 100.

***In vitro* cytotoxic study**

The cytotoxicity (CC_50_) of ART-isatin hybrids **4a-k** and reference drugs artemisinin, dihydroartemisinin, and adriamycin were examined by the MTT assay in MCF-10A cells. The compounds were dissolved in DMSO with different concentrations. The MCF-10A cells were maintained in culture medium at 37 ^o^C under 5% CO_2_ atmosphere. Cells were seeded in 96-well plates (1×10^4^ cell per well) and allowed to recover for 24 h. After 72 h of exposure, cells were harvested, and cell viability was assessed by MTT assay.

**Statistical analysis**

The statistical analysis was performed using Excel © and IC_50_ values were estimated using GraphpadPrism5 software (Hearne Scientific Software). The experiments were performed in duplicate and the statistical significance was calculated using the student’s t-test. A p-value of less than 0.05 was used to estimate the significance of the observations. A Z-factor was calculated for each 96-well plate and assays having Z-factor above 0.05 were included in the statistical analysis.

**Target Prediction of 4a**

The **4a** compounds were imported into the Swiss Target Prediction database (http://www.swisstargetprediction.ch/) to identify potential targets, and the threshold for predicted targets selected in Swiss Target Prediction was " similarity (0.65 for 2D and 0.85 for 3D)" [2].

**Construction of cross-over genes**

For construction of potential targets- and breast cancer- cross-over genes, we Searched the GeneCards database (https://www.genecards.org/) using "Breast Cancer" as a keyword. The background was set to human genes to obtain breast cancer-related genes in the GeneCards database. The breast cancer-related genes were ranked in descending order of Relevance score and the Top 1000 were selected. VENNY2.1 for obtaining cross-over genes and drawing Venn diagram [3].

**Construction of PPI network, GO enrichment analysis and KEGG enrichment analysis**

The cross-over genes were imported into the STRING database (https://string-db.org/) to create the PPI network, the species was set to Homo sapiens, and the minimum interaction score was set to 0.4 [4]. Cytoscape 3.9.1 is used to visualize and analyze PPI results and calculate closeness centrality, betweenness centrality, and degree value. David Database ( https://david.ncifcrf.gov/ ) is used for gene ontology (GO) and the Kyoto Encyclopedia of Genes and Genomes (KEGG) for enrichment analysis, and RStudio 2023.03.0+386 for visualization of enrichment analysis [5]. All enrichment analyses were ranked in descending order of ratio and performed by selecting the TOP20.

**Molecular docking**

The structures of Ligands were created by ChemDraw 2021, and then the structures were optimized by the LigPrep tool. The sequence of proteins was retrieved from the RCSB database (https://www.rcsb.org/). Molecular docking of the proteins and ligands were carried out by using Maestro 11.5 version. Firstly, the protein was optimized before docking using the Protein Preparation Wizard, and the ligand was prepared with LigPrep tool. Further, partial atomic charges attribution, protonation states generation at pH 7 ± 2.0 and energy minimization were achieved using OPLS-2005 force field. To test the docking parameters, all ligands were docked into the catalytic pocket of the protein using Grid-Based Ligand Docking with Energetics (Glide v11.5, Schrödinger) in 'extra precision' mode without applying any constraints. PyMol2.4 was used to visualize the composite PDB format file.

**Molecular dynamics**

The molecular dynamics (MD) simulations were carried out by GROMACS 2020.3 software. The simulation box size was optimized with the distance between each atom of the protein and the box greater than 1.0 nm. Then, fill the box with water molecules based on a density of 1. To make the simulation system electrically neutral, the water molecules were replaced with Cl- and Na+ ions. Following the steepest descent method, energy optimization of 5.0×10^4^ steps was performed to minimize the energy consumption of the entire system, and finally to reduce the unreasonable contact or atom overlap in the entire system. After energy minimization, first-phase equilibration was performed with the NVT ensemble at 300 K for 100 ps to stabilize the temperature of the system. Second-phase equilibration was simulated with the NPT ensemble at 1 bar and 100 ps. The primary objective of the simulation is to optimize the interaction between the target protein and the solvent and ions so that the simulation system is fully pre-equilibrated. All MD simulations were performed for 50 ns under an isothermal and isostatic ensemble with a temperature of 300 K and a pressure of 1 atmosphere. The temperature and pressure were controlled by the V-rescale and Parrinello-Rahman methods, respectively, and the temperature and pressure coupling constants were 0.1 and 0.5 ps, respectively. Lennard-Jones function was used to calculate the Van der Waals force, and the nonbond truncation distance was set to 1.4 nm. The bond length of all atoms was constrained by the LINCS algorithm. The long-range electrostatic interaction was calculated by the Particle Mesh-Ewald method with the Fourier spacing 0.16 nm. The gmx_mmpbsa script determines the binding energy of all protein-ligand complexes in the equilibrium phase [6].

**Characterization**

**Analytical data and analytical spectra of hybrids 4a-4k [1].**

1-(2-(((3R,5aS,6R,8aS,9R,12R,12aR)-3,6,9-trimethyldecahydro-12H-3,12-epoxy[1,2]dioxepino[4,3-i]isochromen-10-yl)oxy)ethyl)indoline-2,3-dione (**4a**)

Red solid, yield: 53%. ^1^H NMR (600 MHz, CDCl_3_) δ 0.68 (d, *J* = 4.0 Hz, 2H), 0.91-1.07 (m, 5H), 1.20-1.35 (m, 3H), 1.37-1.56 (m, 5H), 1.67-1.72 (m, 1H), 1.88-2.06 (m, 2H), 2.25-2.42 (m, 2H), 3.78-4.15 (m, 4H), 4.46 (d, *J* = 4.0 Hz, 1H), 5.34 (s, 1H), 7.06-7.11 (m, 1H), 7.44 (d, J = 4.0 Hz, 1H), 7.55-7.60 (m, 2H). ^13^C NMR (150 MHz, CDCl_3_) 183.69, 183.36, 158.60, 158.40, 152.10, 151.44, 138.52, 138.13, 125.19, 124.87, 123.58, 123.51, 117.56, 117.36, 112.42, 111.15, 104.37, 103.27, 103.07, 101.29, 91.16, 89.12, 81.50, 80.38, 67.70, 65.83, 51.66, 51.59, 45.24, 41.48, 39.76, 37.38, 37.24, 36.29, 34.15, 32.63, 26.01, 24.69, 22.10, 20.25, 19.97, 12.33. HRMS-ESI: m/z Calcd for C_25_H_34_N_2_O_7_ [M+NH_4_]^+^: 475.2435; Found: 475.2408.


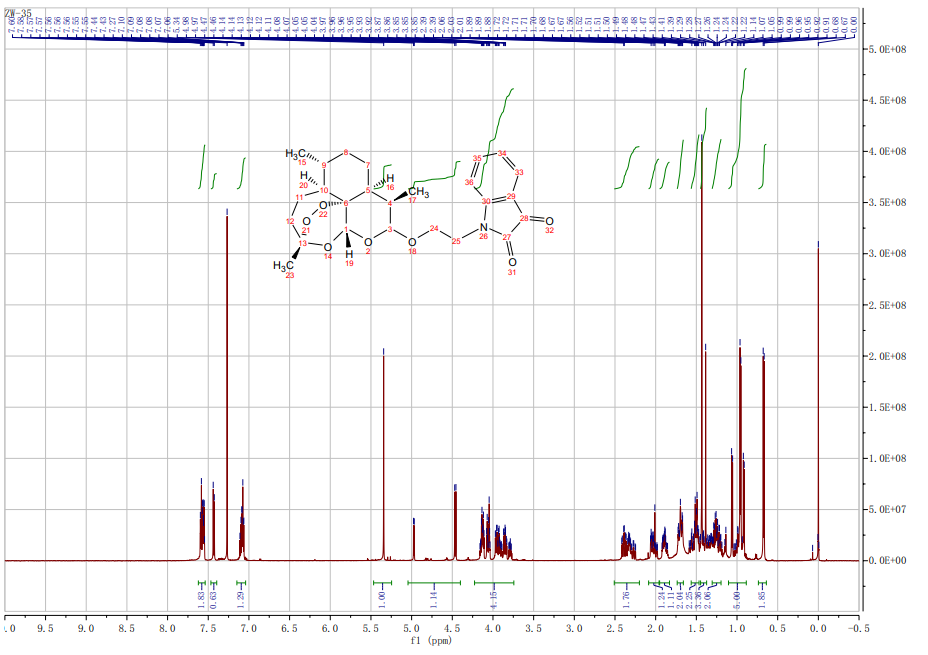


**Figure S1.** The ^1^H NMR spectra of **4a**.


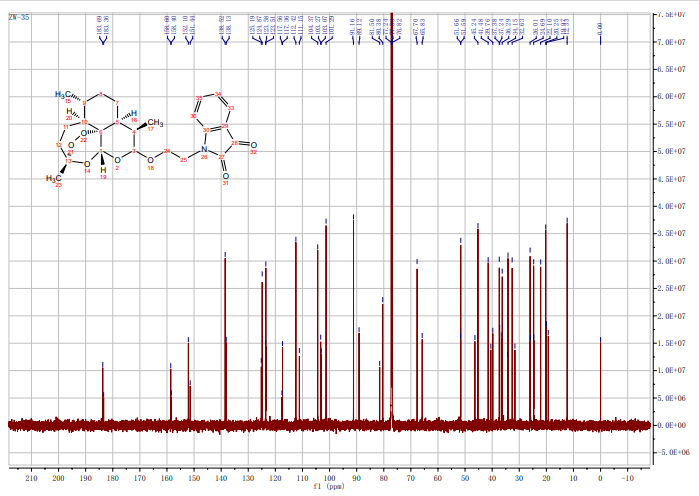


**Figure S2.** The ^13^C NMR spectra of **4a**.


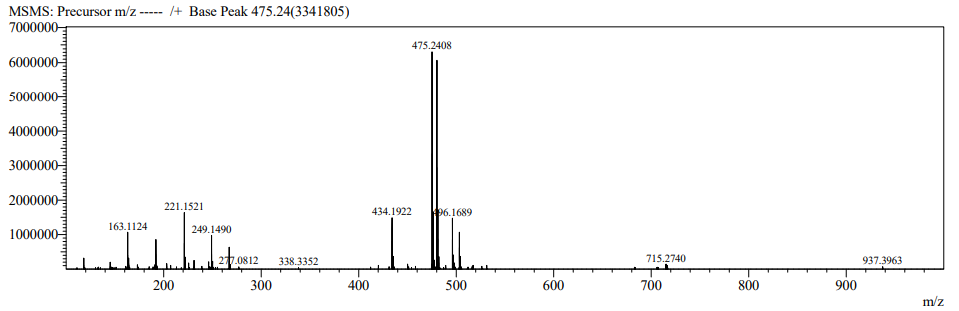


**Figure S3.** The HRMS spectra of **4a**.

5-fluoro-1-(2-(((3R,5aS,6R,8aS,9R,12R,12aR)-3,6,9-trimethyldecahydro-12H-3,12-epoxy[1,2]dioxepino[4,3-i]isochromen-10-yl)oxy)ethyl)indoline-2,3-dione (**4b**)

Red solid, yield: 48%. ^1^H NMR (600 MHz, CDCl_3_) 0.77-0.97 (m, 7H), 1.08-1.21 (m, 2H), 1.38-1.45 (m, 5H), 1.48-1.53 (m, 2H), 1.55-1.59 (m, 1H), 1.83-1.86 (m, 1H), 1.99-2.05 (m, 1H), 2.30-2.36 (m, 1H), 2.57-2.63 (m, 1H), 3.65-3.68 (m, 1H), 3.90-3.94 (m, 1H), 4.02-4.06 (m, 1H), 4.14-4.18 (m, 1H), 4.76 (d, *J* = 2.0 Hz, 1H), 5.22 (s, 1H), 6.95-6.97 (m, 1H), 7.29-7.32 (m, 2H). ^13^C NMR (150 MHz, CDCl_3_) 182.80, 160.07, 158.09, 147.10, 124.54, 124.38, 118.10, 112.39, 112.23, 111.93, 111.88, 104.22, 102.53, 87.81, 80.81, 64.79, 60.43, 53.32, 44.05, 43.09, 40.28, 37.47, 36.29, 34.40, 30.62, 26.06, 24.62, 24.53, 20.21, 12.80. HRMS-ESI: m/z Calcd for C_25_H_30_FNO_7_Na [M+Na]^+^: 498.1899; Found: 498.1897.


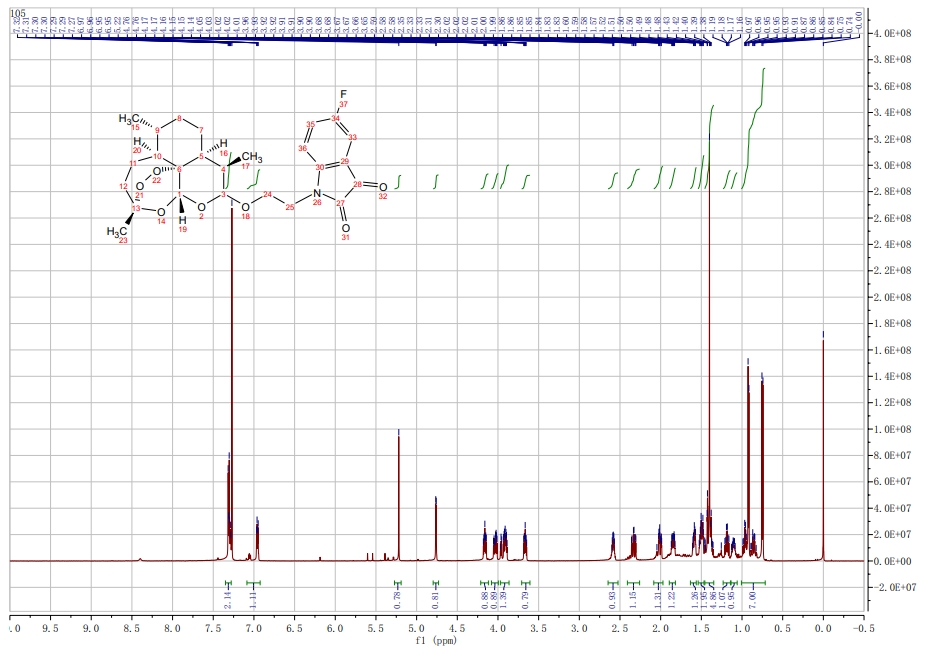


**Figure S4.** The ^1^H NMR spectra of **4b**.


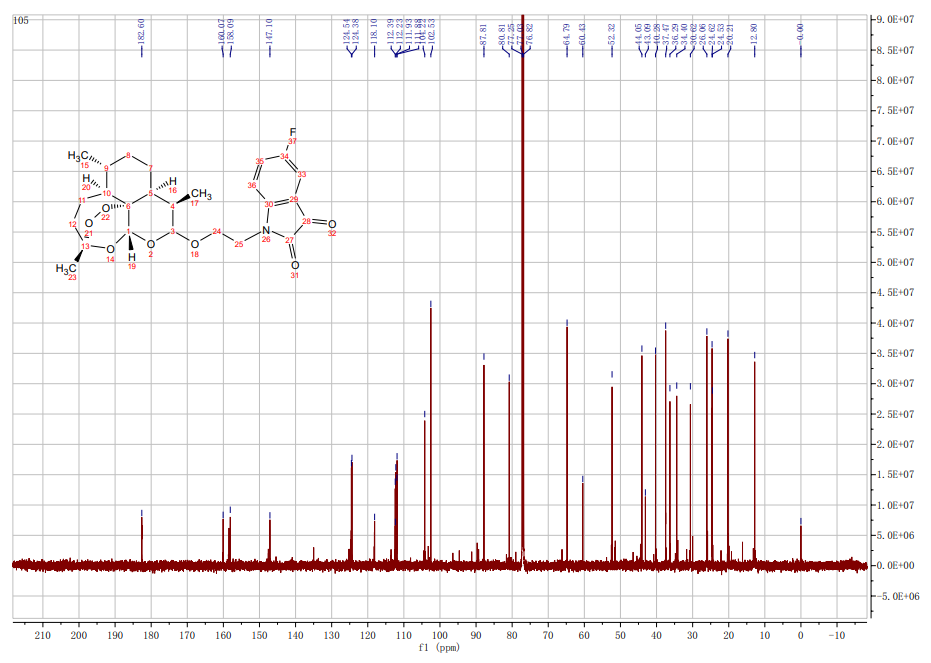


**Figure S5.** The ^13^C NMR spectra of **4b**.


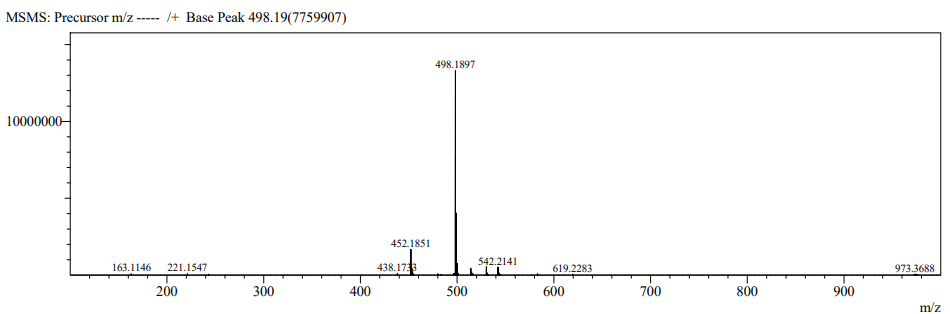


**Figure S6.** The HRMS spectra of **4b**.

5-methoxy-1-(2-(((3R,5aS,6R,8aS,9R,12R,12aR)-3,6,9-trimethyldecahydro-12H-3,12-epoxy[1,2]dioxepino[4,3-i]isochromen-10-yl)oxy)ethyl)indoline-2,3-dione (**4c**)

Red solid, yield: 51%. ^1^H NMR (600 MHz, CDCl_3_) δ 0.76 (d, J = 7.4 Hz, 3H), 0.88 (d, J = 6.4 Hz, 3H), 0.94 – 1.05 (m, 1H), 1.13 (td, J = 11.5, 6.6 Hz, 1H), 1.30 – 1.39 (m, 3H), 1.40 (s, 3H), 1.41 – 1.50 (m, 2H), 1.50 – 1.60 (m, 2H), 1.80 (ddt, J = 13.5, 6.7, 3.5 Hz, 2H), 1.97 (ddd, J = 14.5, 4.6, 3.1 Hz, 1H), 2.30 (td, J = 14.1, 3.9 Hz, 1H), 2.55 (dt, J = 7.8, 4.2 Hz, 1H), 3.62 (dt, J = 10.3, 5.1 Hz, 1H), 3.81 (s, 3H), 3.86 (dt, J = 14.5, 5.0 Hz, 1H), 4.09 (ddd, J = 14.5, 7.5, 4.7 Hz, 1H), 4.21 (ddd, J = 10.4, 7.6, 4.4 Hz, 1H), 4.76 (d, J = 3.4 Hz, 1H), 5.13 (s, 1H), 6.85 (d, J = 8.6 Hz, 1H), 6.93 (dd, J = 8.6, 2.7 Hz, 1H), 7.73 (d, J = 2.6 Hz, 1H). ^13^C NMR (150 MHz, CDCl_3_) 158.32, 156.13, 124.68, 111.72, 109.24, 107.03, 104.16, 104.10, 102.45, 102.13, 87.81, 80.88, 80.85, 64.71, 55.96, 52.42, 52.35, 44.10, 40.08, 37.40, 37.22, 36.35, 36.32, 34.42, 30.71, 30.67, 26.12, 26.09, 24.62, 24.49, 20.23, 12.82. HRMS-ESI: m/z Calcd for C_26_H_33_NO_8_Na [M+Na]^+^: 510.2098; Found: 510.2084.


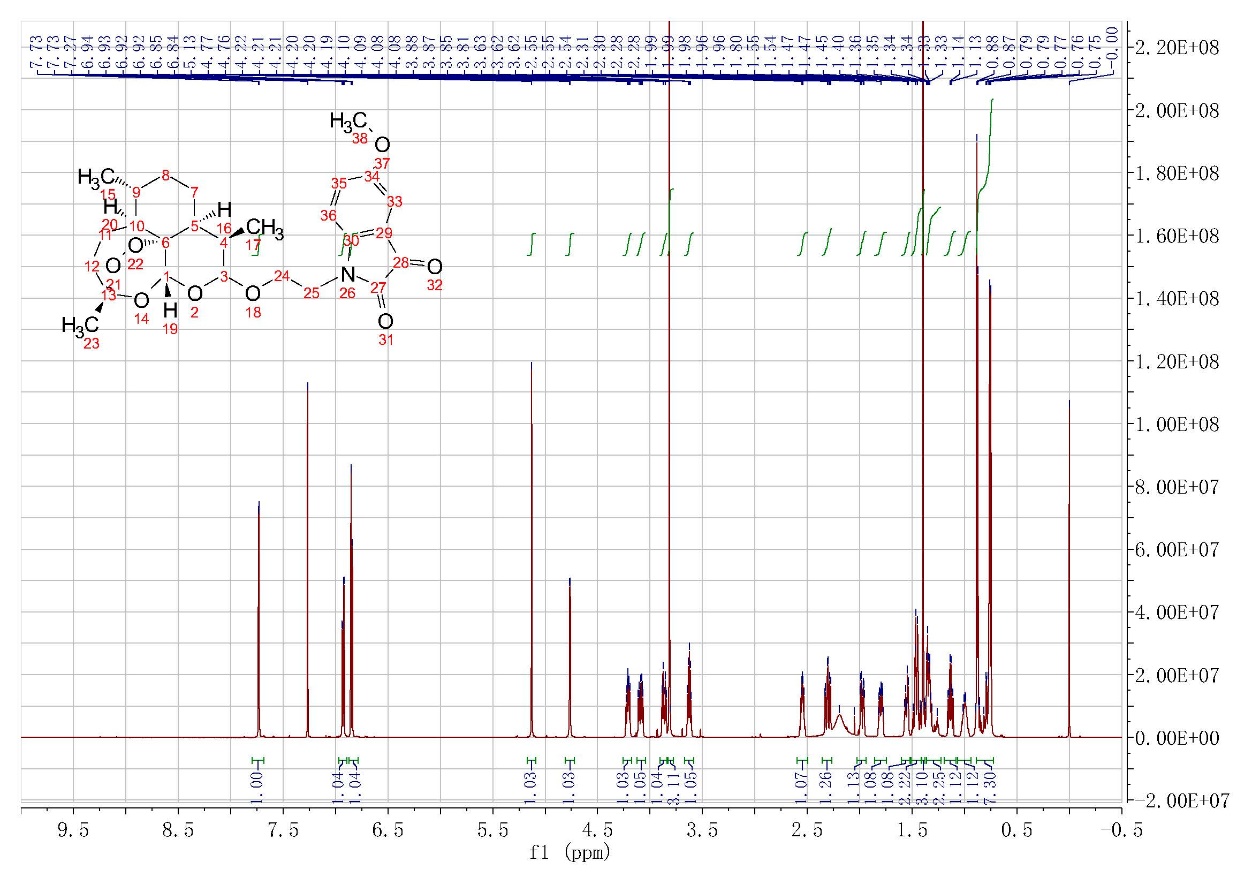


**Figure S7.** The ^1^H NMR spectra of **4c**.


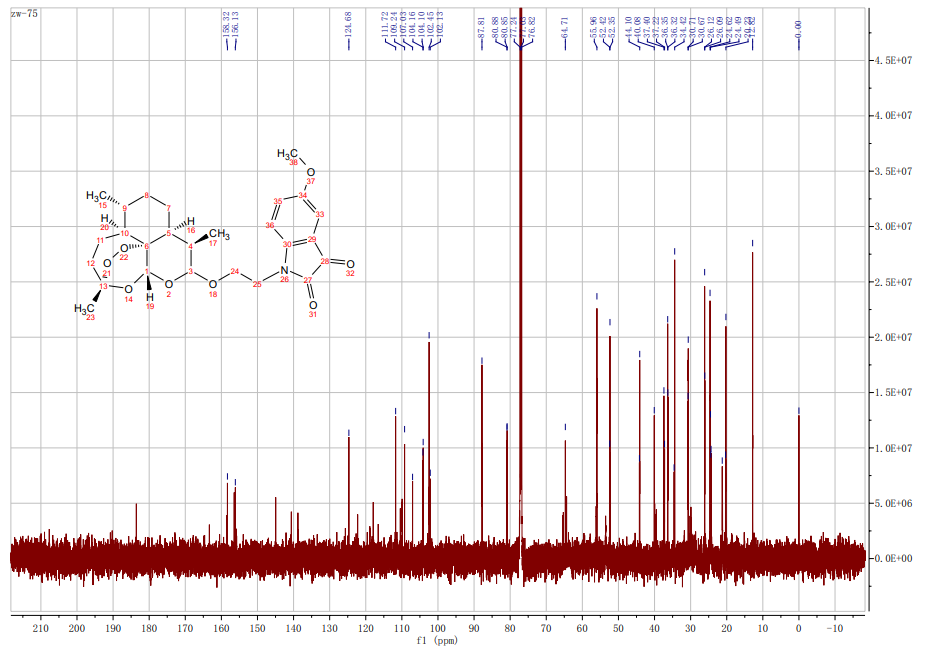


**Figure S8.** The ^13^C NMR spectra of **4c**.


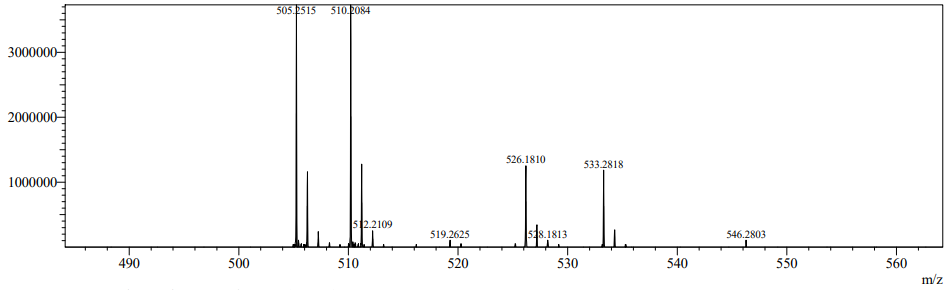


**Figure S9.** The HRMS spectra of **4c**.

3-(methoxyimino)-1-(2-(((3R,5aS,6R,8aS,9R,12R,12aR)-3,6,9-trimethyldecahydro-12H-3,12-epoxy[1,2]dioxepino[4,3-i]isochromen-10-yl)oxy)ethyl)indolin-2-one (**4d**)

Yellow solid, yield: 86%. ^1^H NMR (600 MHz, CDCl_3_) δ 0.62 (d, *J* = 4.0 Hz, 2H), 0.90-1.07 (m, 5H), 1.18-1.42 (m, 5H), 1.44-1.59 (m, 3H), 1.64-1.72 (m, 2H), 1.83-2.06 (m, 2H), 2.25-2.42 (m, 2H), 3.73-3.81 (m, 1H), 3.92-3.96 (m, 2H), 4.05-4.15 (m, 1H), 4.29 (s, 1H, NOCH_3_), 4.43 (d, *J* = 8.0 Hz, 1H), 5.32 (s, 1H), 6.95-7.08 (m, 2H), 7.38 (t, J = 4.0 Hz, 1H), 7.92 (d, J = 4.0 Hz, 1H). ^13^C NMR (150 MHz, CDCl_3_) 163.79, 144.76, 143.83, 134.99, 132.54, 132.47, 127.96, 127.49, 123.12, 122.78, 122.72, 115.52, 110.55, 109.55, 109.00, 104.55, 104.33, 103.27, 101.01, 91.18, 89.69, 88.85, 80.39, 67.54, 65.64, 64.85, 64.75, 64.68, 60.76, 51.81, 51.62, 51.44, 45.29, 44.45, 40.81, 37.49, 37.36, 36.31, 36.24, 34.18, 34.11, 32.62, 30.00, 26.03, 24.70, 24.42, 22.12, 20.26, 16.19, 12.26. HRMS-ESI: m/z Calcd for C_26_H_34_N_2_O_7_Na [M+Na]^+^: 509.2258; Found: 509.2240.


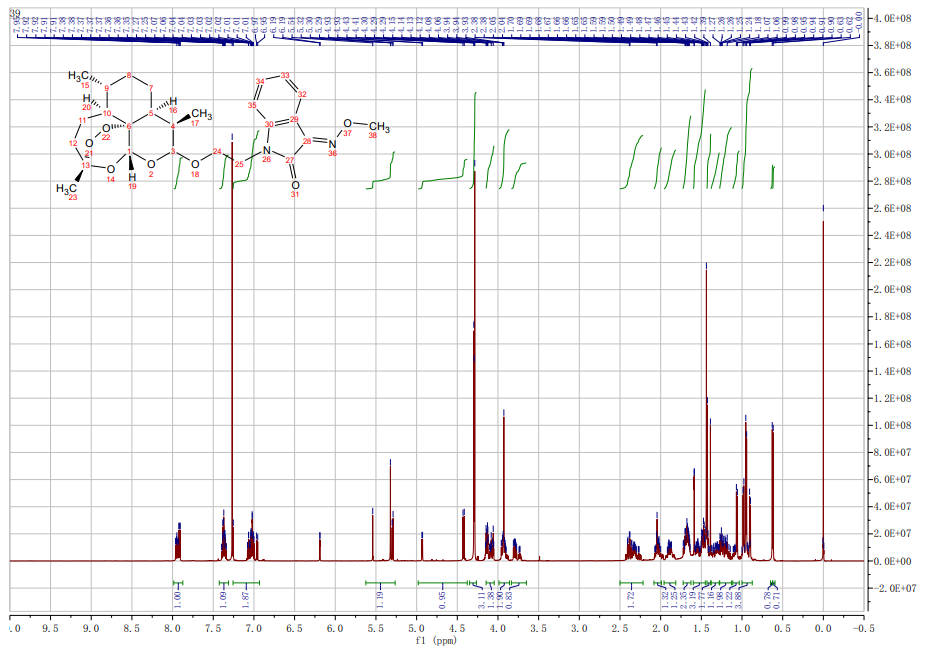


**Figure S10.** The ^1^H NMR spectra of **4d**.


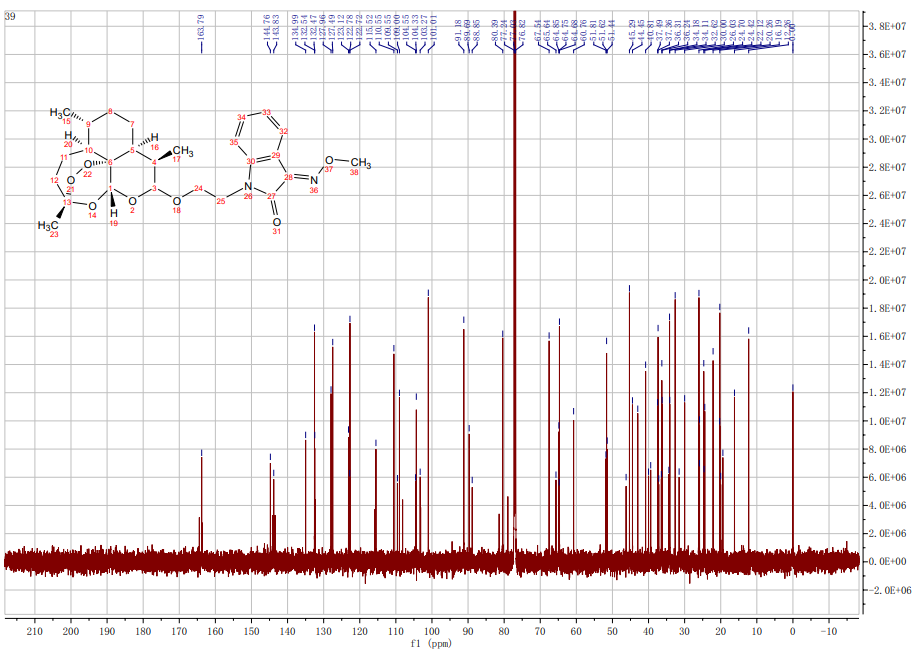


**Figure S11.** The ^13^C NMR spectra of **4d**.


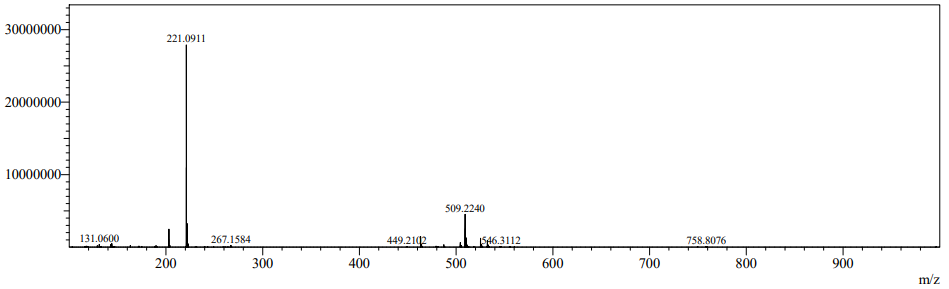


**Figure S12.** The HRMS spectra of **4d**.

(E)-5-fluoro-3-(methoxyimino)-1-(2-(((3R,5aS,6R,8aS,9R,12R,12aR)-3,6,9-trimethyldecahydro-12H-3,12-epoxy[1,2]dioxepino[4,3-i]isochromen-10-yl)oxy)ethyl)indolin-2-one (**4e**)

Yellow solid, yield: 94%. ^1^H NMR (600 MHz, CDCl_3_) 0.72-0.96 (m, 7H), 1.03-1.08 (m, 1H), 1.04-1.18 (m, 1H), 1.36-1.48 (m, 7H), 1.54-1.57 (m, 1H), 1.81-1.84 (m, 1H), 1.98-2.01 (m, 1H), 2.29-2.35 (m, 1H), 2.54-2.57 (m, 1H), 3.60-3.62 (m, 1H), 3.82-3.88 (m, 2H), 4.05-4.10 (m, 1H), 4.15-4.18 (m, 1H), 4.75 (d, *J* = 2.0 Hz, 1H), 5.14 (s, 1H), 6.85 (dd, *J* = 8.0, 4.0 Hz, 1H), 7.08 (dd, *J* = 8.0, 2.0 Hz, 1H), 7.70 (dd, *J* = 8.0, 2.0 Hz, 1H). ^13^C NMR (150 MHz, CDCl_3_) 163.34, 159.52, 157.93, 143.10, 140.00, 118.55, 118.39, 116.17, 115.40, 115.23, 109.83, 109.78, 104.10, 102.29, 87.74, 80.85, 65.10, 65.05, 64.74, 60.78, 52.36, 44.13, 43.10, 39.89, 37.38, 36.33, 34.49, 30.66, 26.09, 24.60, 24.49, 20.23, 12.77. HRMS-ESI: m/z Calcd for C_26_H_33_FN_2_O_7_Na [M+Na]^+^: 527.2164; Found: 527.2151.


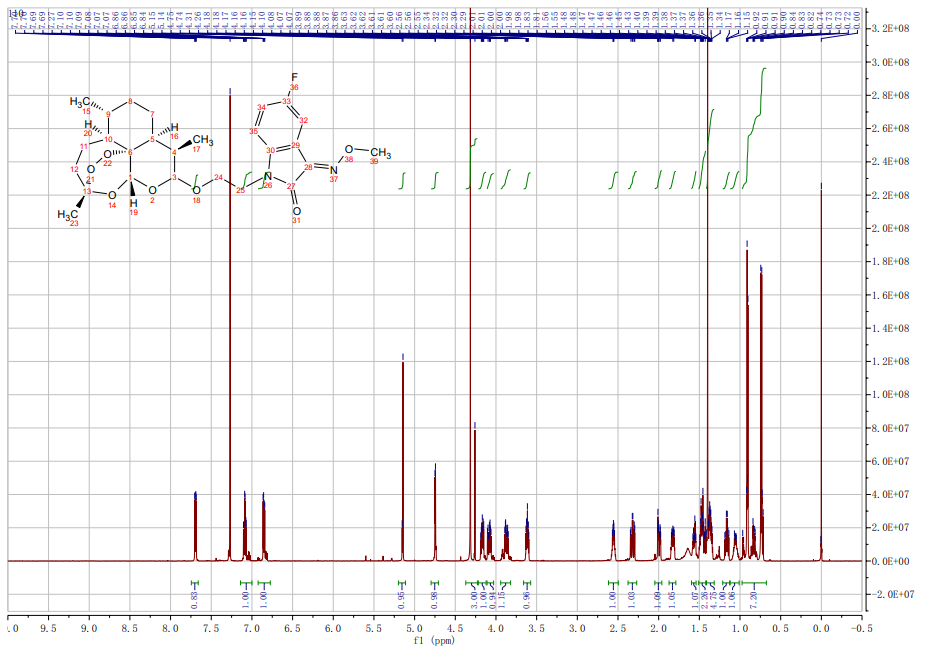


**Figure S13.** The ^1^H NMR spectra of **4e**.


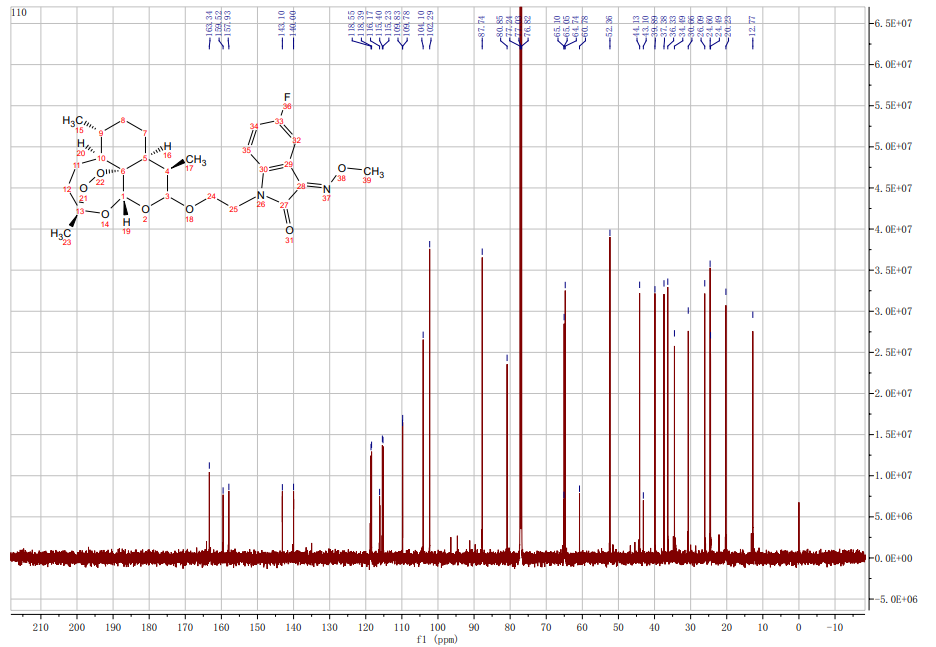


**Figure S14.** The ^13^C NMR spectra of **4e**.


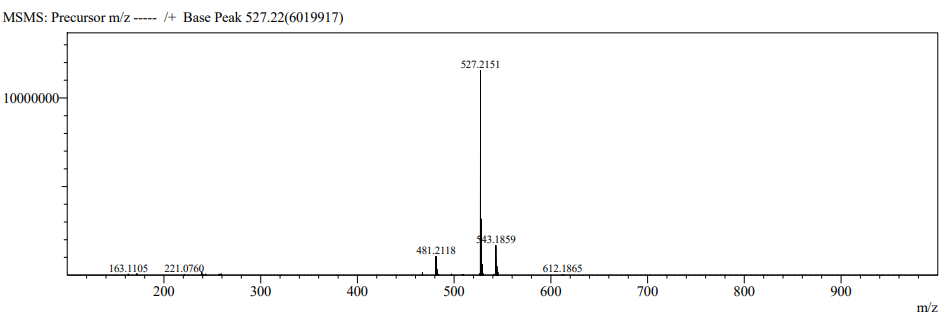


**Figure S15.** The HRMS spectra of **4e**.

5-methoxy-3-(methoxyimino)-1-(2-(((3R,5aS,6R,8aS,9R,12R,12aR)-3,6,9-trimethyldecahydro-12H-3,12-epoxy[1,2]dioxepino[4,3-i]isochromen-10-yl)oxy)ethyl)indolin-2-one (**4f**)

Yellow solid, yield: 81%. ^1^H NMR (600 MHz, CDCl_3_) 0.73-0.91 (m, 7H), 1.02-1.05 (m, 1H), 1.13-1.17 (m, 1H), 1.32-1.38 (m, 2H), 1.40 (s, 3H), 1.43-1.52 (m, 2H), 1.51-1.59 (m, 1H), 1.80-1.84 (m, 1H), 1.97-2.01 (m, 1H), 2.29-2.34 (m, 1H), 2.53-2.56 (m, 1H), 3.59-3.62 (m, 1H), 3.81 (s, 3H), 3.83-3.87 (m, 1H), 4.05-4.09 (m, 1H), 4.15-4.19 (m, 1H), 4.30 (s, 3H), 4.74 (d, *J* = 2.0 Hz, 1H), 5.13 (s, 1H), 6.82 (d, *J* = 4.0 Hz, 1H), 6.92 (dd, *J* = 4.0, 2.0 Hz, 1H), 7.56 (d, *J* = 2.0 Hz, 1H). ^13^C NMR (150 MHz, CDCl_3_) 163.48, 155.73, 143.77, 137.66, 117.41, 116.24, 114.17, 109.68, 104.05, 102.23, 87.74, 80.90, 64.84, 64.64, 55.99, 52.38, 44.18, 39.72, 37.30, 36.36, 34.51, 30.70, 26.11, 24.60, 24.45, 20.25, 12.80. HRMS-ESI: m/z Calcd for C_27_H_36_N_2_O_8_Na [M+Na]^+^: 539.2364; Found: 539.2351.


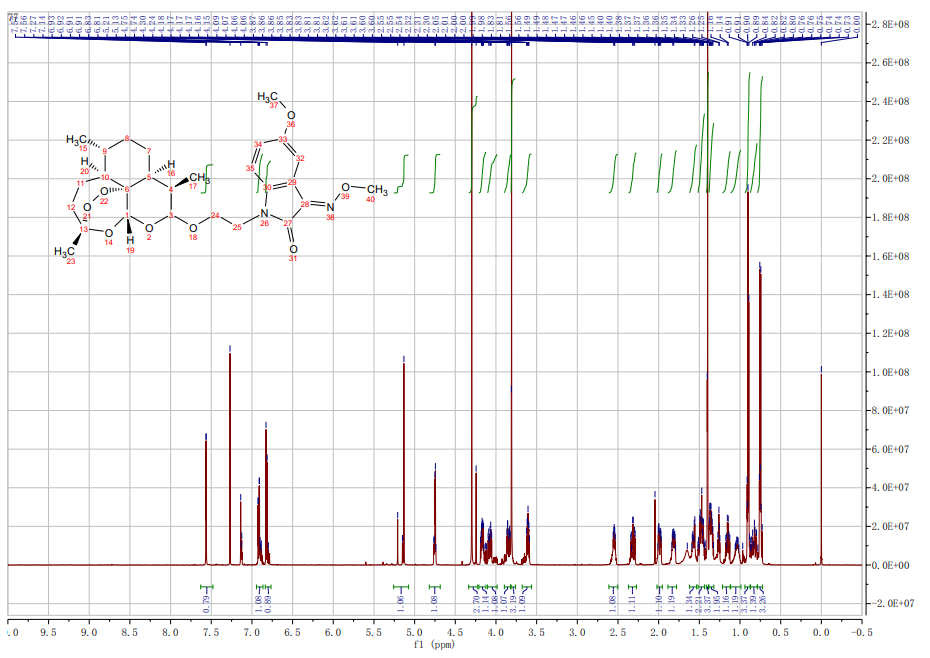


**Figure S16.** The ^1^H NMR spectra of **4f**.


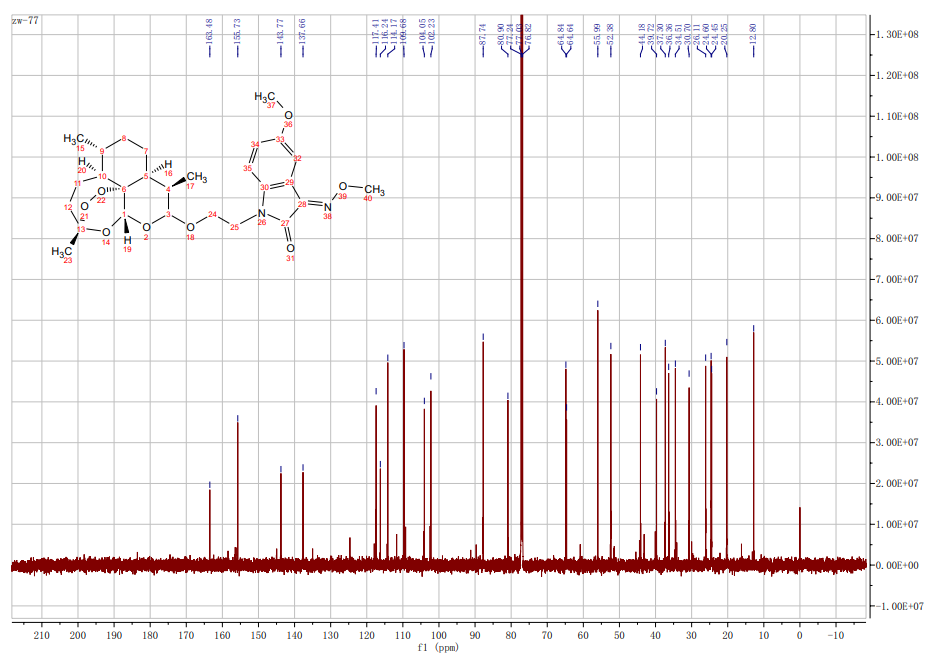


**Figure S17.** The ^13^C NMR spectra of **4f**.


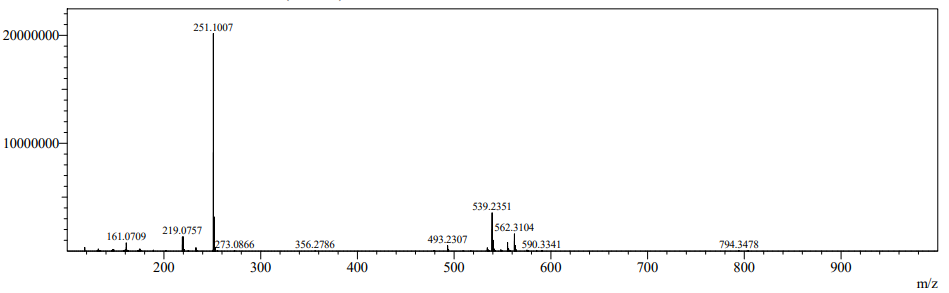


**Figure S18.** The HRMS spectra of **4f**.

3-(ethoxyimino)-1-(2-(((3R,5aS,6R,8aS,9R,12R,12aR)-3,6,9-trimethyldecahydro-12H-3,12-epoxy[1,2]dioxepino[4,3-i]isochromen-10-yl)oxy)ethyl)indolin-2-one (**4g**)

Yellow solid, yield: 65%. ^1^H NMR (600 MHz, CDCl_3_) δ 0.62 (d, J = 4.0 Hz, 2H), 0.84-1.07 (m, 5H), 1.19-1.39 (m, 4H), 1.44-1.54 (m, 6H), 1.65-1.70 (m, 2H), 1.82-2.05 (m, 2H), 2.31-2.41 (m, 2H), 3.73-3.95 (m, 2H), 4.06-4.16 (m, 2H), 4.42 (d, *J* = 2.0 Hz, 1H), 4.46 (q, *J* = 2.0 Hz, 2H, NOCH2CH3), 5.32 (s, 1H), 7.00-7.25 (m, 2H), 7.38 (t, J = 4.0 Hz, 1H), 7.94 (d, J = 4.0 Hz, 1H). ^13^C NMR (150 MHz, CDCl_3_) 163.92, 144.64, 143.67, 132.36, 127.40, 122.66, 115.63, 110.47, 104.33, 100.98, 91.18, 80.39, 67.55, 51.63, 45.29, 40.77, 37.36, 36.32, 34.19, 32.63, 26.04, 25.91, 24.70, 22.12, 20.26, 19.41, 14.71, 12.26. HRMS-ESI: m/z Calcd for C_27_H_36_N_2_O_7_Na [M+Na]^+^: 523.2415; Found: 523.2411.


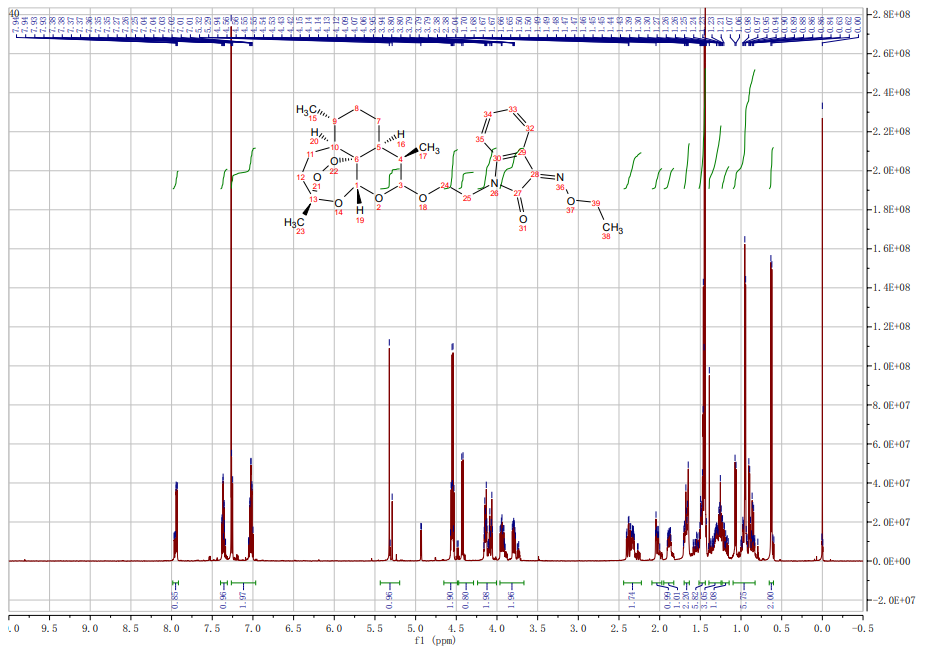


**Figure S19.** The ^1^H NMR spectra of **4g**.


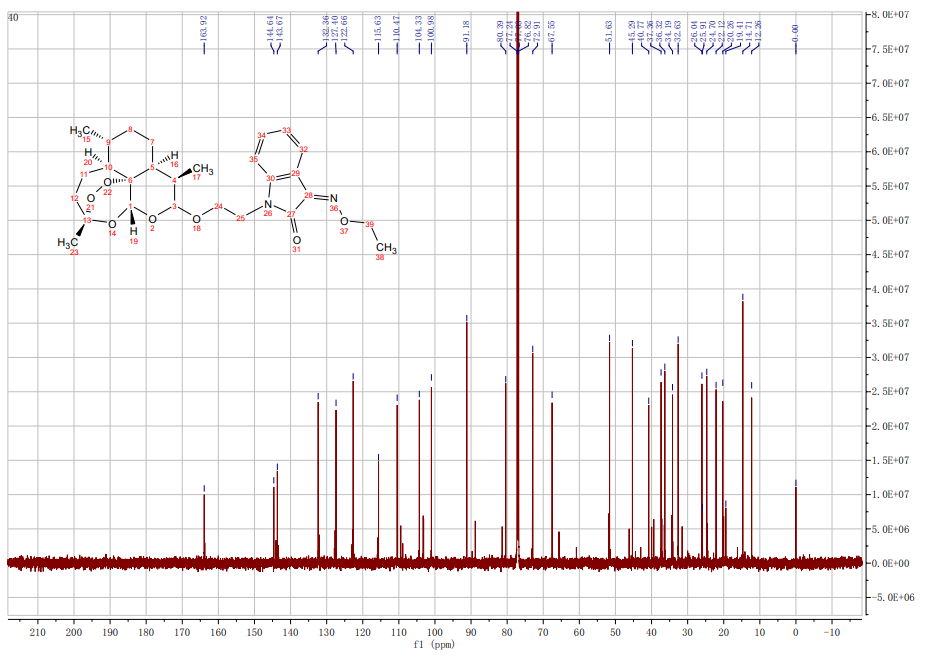


**Figure S20.** The ^13^C NMR spectra of **4g**.


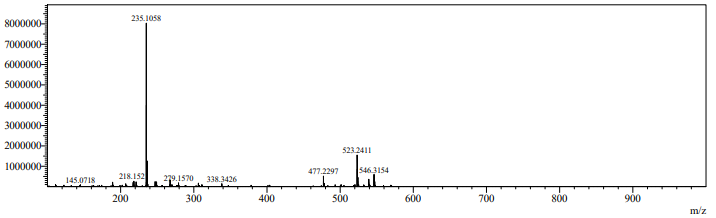


**Figure S21.** The HRMS spectra of **4g**.

3-(ethoxyimino)-5-fluoro-1-(2-(((3R,5aS,6R,8aS,9R,12R,12aR)-3,6,9-trimethyldecahydro-12H-3,12-epoxy[1,2]dioxepino[4,3-i]isochromen-10-yl)oxy)ethyl)indolin-2-one (**4h**)

Yellow solid, yield: 88%. ^1^H NMR (600 MHz, CDCl_3_) 0.72-0.97 (m, 7H), 1.01-1.05 (m, 1H), 1.13-1.18 (m, 1H), 1.36-1.48 (m, 10H), 1.55-1.58 (m, 1H), 1.81-1.84 (m, 1H), 1.97-2.01 (m, 1H), 2.29-2.35 (m, 1H), 2.54-2.57 (m, 1H), 3.59-3.63 (m, 1H), 3.85-3.92 (m, 1H), 4.07-4.11 (m, 1H), 4.15-4.19 (m, 1H), 4.57 (d, *J* = 8.0 Hz, 2H), 4.75 (d, *J* = 2.0 Hz, 1H), 5.14 (s, 1H), 6.85 (dd, *J* = 8.0, 4.0 Hz, 1H), 7.08 (dd, *J* = 8.0, 2.0 Hz, 1H), 7.70 (dd, *J* = 8.0, 2.0 Hz, 1H). ^13^C NMR (150 MHz, CDCl_3_) 163.48, 159.52, 157.92, 142.93, 139.89, 118.34, 118.19, 116.30, 115.29, 115.11, 109.76, 109.70, 104.10, 102.28, 87.73, 80.85, 73.41, 64.73, 52.36, 44.14, 43.10, 39.85, 37.37, 36.34, 34.49, 30.67, 26.10, 24.61, 24.49, 20.23, 14.70, 12.78. HRMS-ESI: m/z Calcd for C_27_H_34_FN_2_O_7_Na [M+Na]^+^: 541.2321; Found: 541.2307.


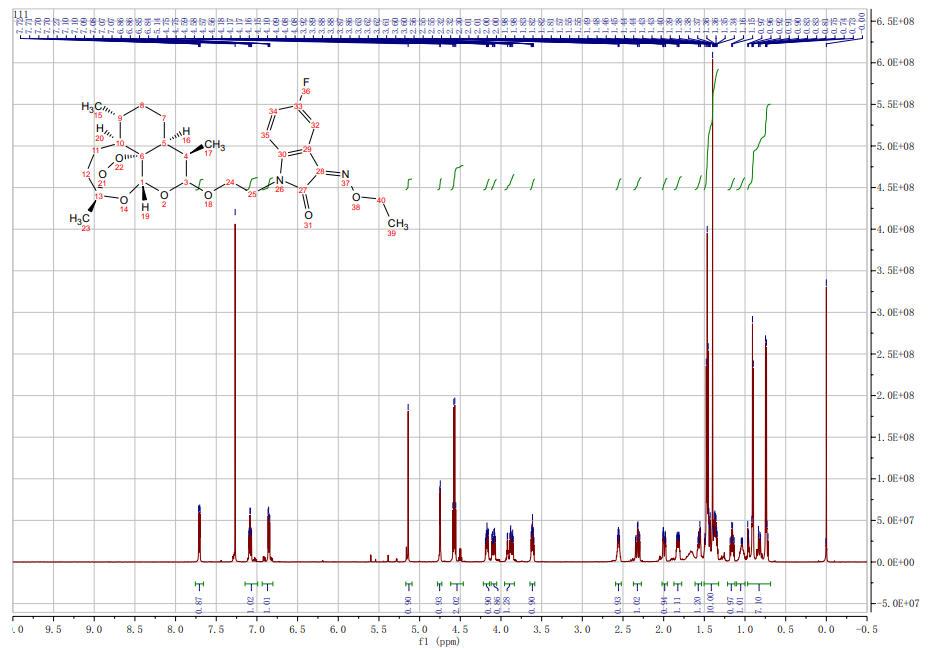


**Figure S22.** The ^1^H NMR spectra of **4h**.


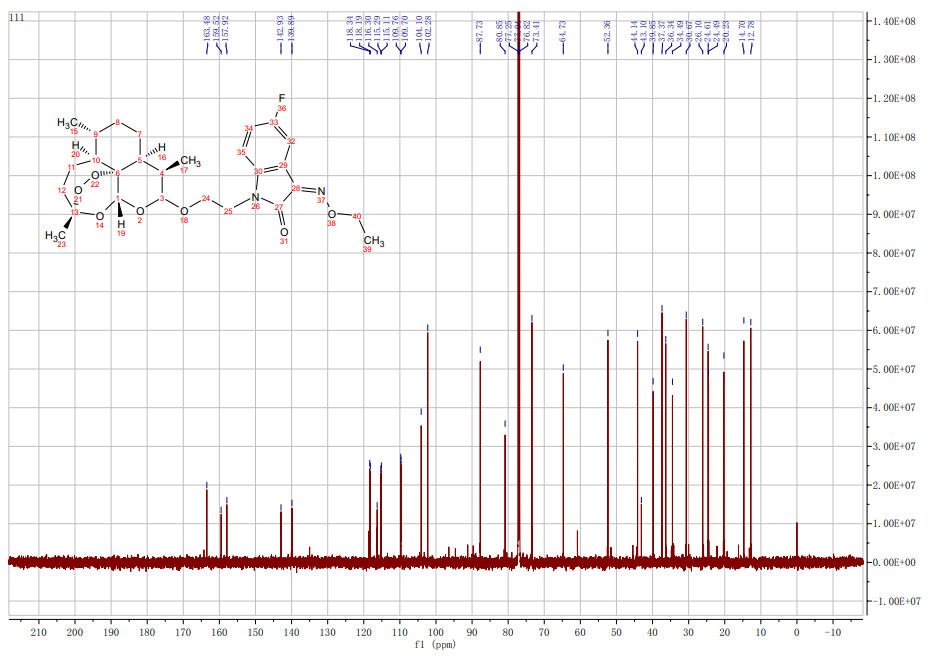


**Figure S23.** The ^13^C NMR spectra of **4h**.


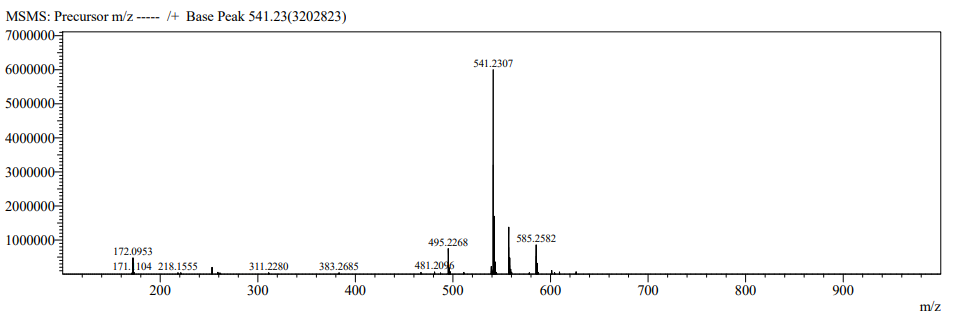


**Figure S24.** The HRMS spectra of **4h**.

3-(ethoxyimino)-5-methoxy-1-(2-(((3R,5aS,6R,8aS,9R,12R,12aR)-3,6,9-trimethyldecahydro-12H-3,12-epoxy[1,2]dioxepino[4,3-i]isochromen-10-yl)oxy)ethyl)indolin-2-one (**4i**)

Yellow solid, yield: 80%. ^1^H NMR (600 MHz, CDCl_3_) 0.75-0.91 (m, 7H), 1.02-1.05 (m, 1H), 1.13-1.16 (m, 1H), 1.34-1.38 (m, 2H), 1.40 (s, 3H), 1.43-1.49 (m, 5H), 1.56-1.59 (m, 1H), 1.80-1.82 (m, 1H), 1.97-2.00 (m, 1H), 2.29-2.34 (m, 1H), 2.53-2.56 (m, 1H), 3.60-3.62 (m, 1H), 3.81 (s, 3H), 3.82-3.87 (m, 1H), 4.07-4.11 (m, 1H), 4.16-4.20 (m, 1H), 4.56 (q, *J* = 4.0 Hz, 3H), 4.75 (d, *J* = 2.0 Hz, 1H), 5.13 (s, 1H), 6.82 (d, *J* = 8.0 Hz, 1H), 6.92 (dd, *J* = 4.0, 2.0 Hz, 1H), 7.60 (d, *J* = 2.0 Hz, 1H). ^13^C NMR (150 MHz, CDCl_3_) 163.63, 155.70, 143.62, 137.53, 116.95, 114.34, 109.57, 104.05, 102.22, 87.74, 80.90, 73.09, 64.61, 55.95, 52.38, 44.19, 39.70, 37.29, 36.36, 34.51, 30.71, 26.13, 24.61, 24.45, 20.26, 14.73, 12.81. HRMS-ESI: m/z Calcd for C_28_H_38_N_2_O_8_Na [M+Na]^+^: 553.2520; Found: 553.2491.


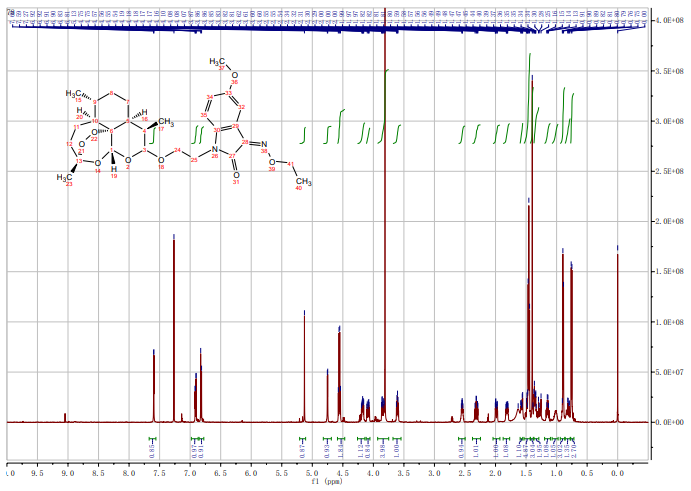


**Figure S25.** The ^1^H NMR spectra of **4i**.


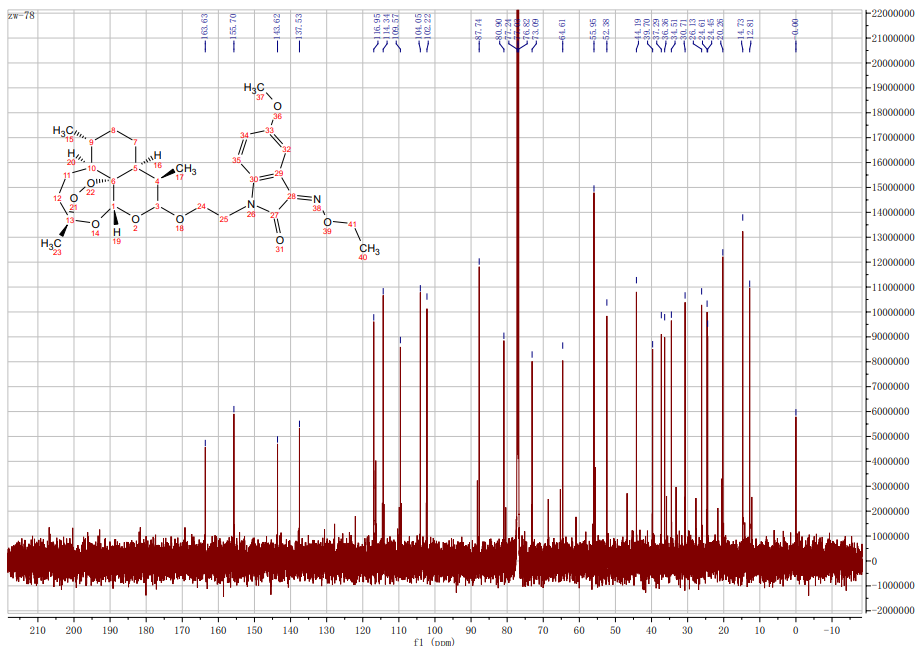


**Figure S26.** The ^13^C NMR spectra of **4i**.


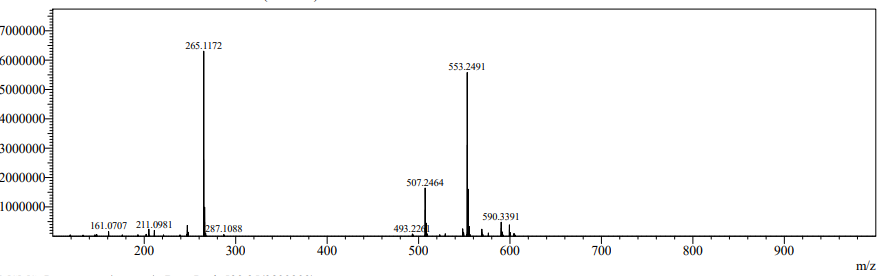


**Figure S27.** The HRMS spectra of **4i**.

3-((benzyloxy)imino)-5-fluoro-1-(2-(((3R,5aS,6R,8aS,9R,12R,12aR)-3,6,9-trimethyldecahydro-12H-3,12-epoxy[1,2]dioxepino[4,3-i]isochromen-10-yl)oxy)ethyl)indolin-2-one (**4j**)

Yellow solid, yield: 69%. ^1^H NMR (600 MHz, CDCl_3_) 0.72-0.89 (m, 7H), 1.02-1.06 (m, 1H), 1.13-1.18 (m, 1H), 1.35-1.47 (m, 7H), 1.54-1.58 (m, 1H), 1.80-1.83 (m, 1H), 1.97-2.01 (m, 1H), 2.29-2.35 (m, 1H), 2.54-2.57 (m, 1H), 3.59-3.63 (m, 1H), 3.85-3.89 (m, 1H), 4.05-4.10 (m, 1H), 4.15-4.18 (m, 1H), 4.74 (d, *J* = 4.0 Hz, 1H), 5.15 (s, 1H), 5.53 (s, 2H), 6.84 (dd, *J* = 8.0, 4.0 Hz, 1H), 7.08 (dd, *J* = 8.0, 2.0 Hz, 1H), 7.37-7.46 (m, 5H), 7.66 (dd, *J* = 8.0, 2.0 Hz, 1H). ^13^C NMR (150 MHz, CDCl_3_) 163.36, 159.51, 157.91, 143.39, 140.01, 135.89, 128.71, 128.68, 128.58, 128.54, 118.58, 118.42, 115.48, 115.31, 109.81, 109.76, 104.11, 102.31, 87.75, 80.85, 79.79, 64.78, 60.79, 52.35, 44.13, 43.11, 39.89, 37.37, 36.33, 34.47, 30.66, 26.10, 24.61, 24.49, 20.22, 12.77. HRMS-ESI: m/z Calcd for C_32_H_37_FN_2_O_7_Na [M+Na]^+^: 603.2477; Found: 603.2450.


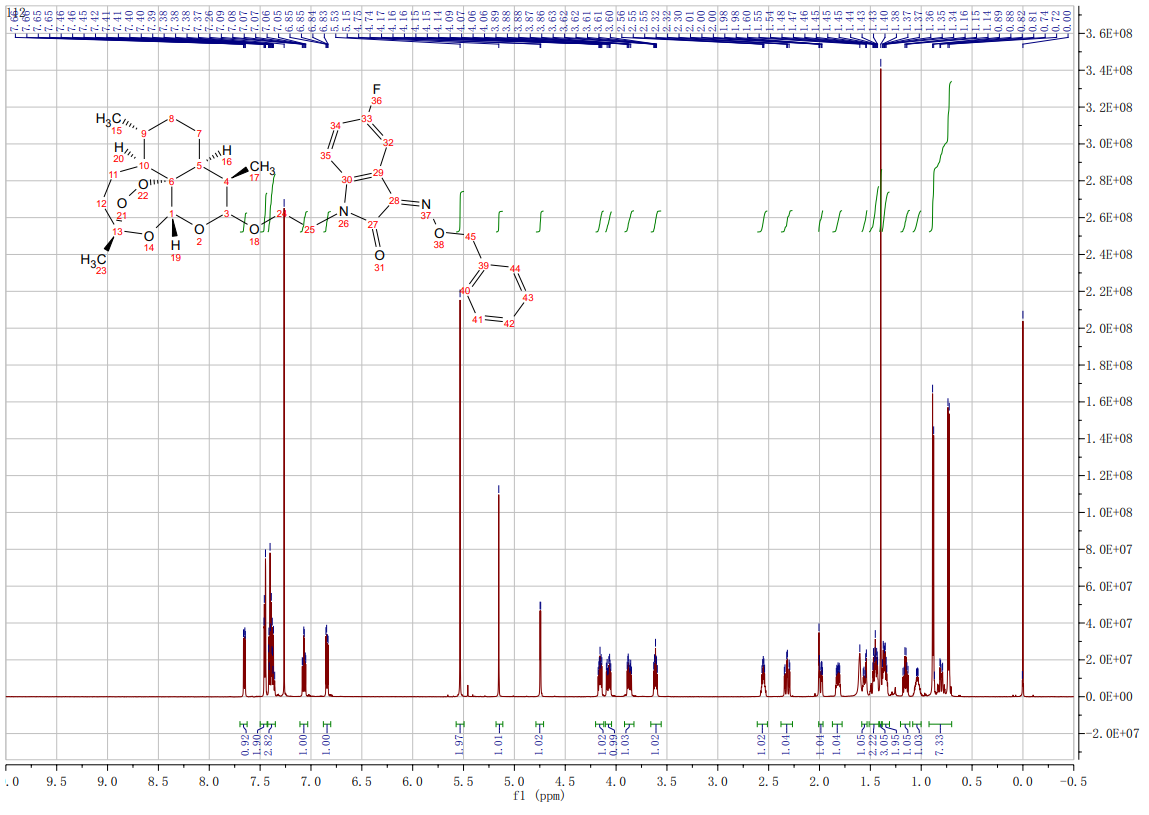


**Figure S28.** The ^1^H NMR spectra of **4j**.


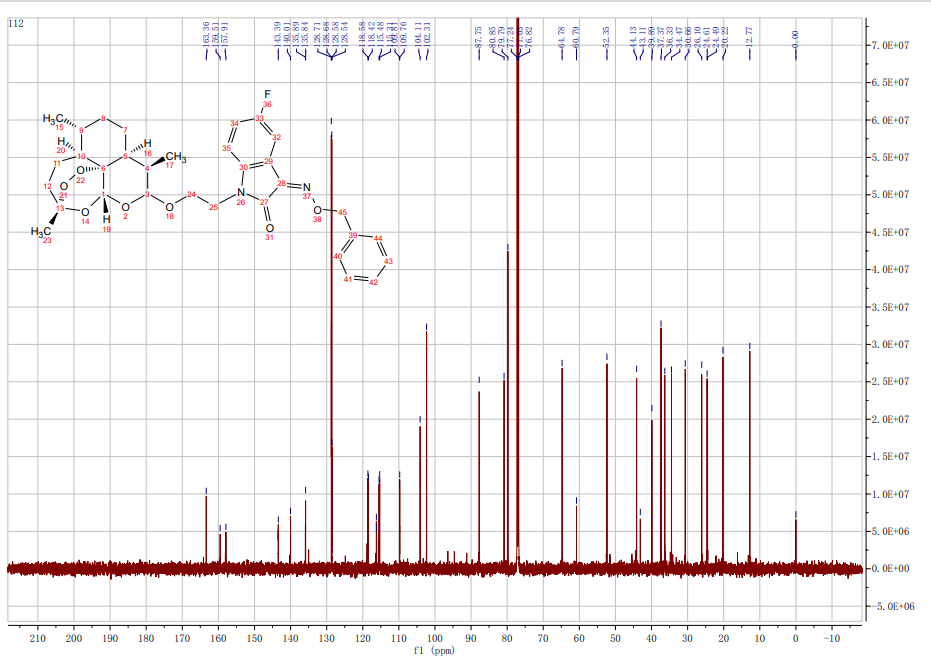


**Figure S29.** The ^13^C NMR spectra of **4j**.


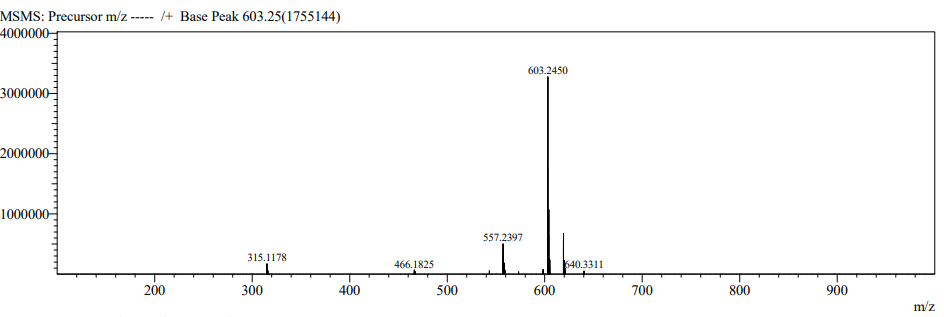


**Figure S30.** The HRMS spectra of **4j**.

(E)-3-((benzyloxy)imino)-5-methoxy-1-(2-(((3R,5aS,6R,8aS,9R,12R,12aR)-3,6,9-trimethyldecahydro-12H-3,12-epoxy[1,2]dioxepino[4,3-i]isochromen-10-yl)oxy)ethyl)indolin-2-one (**4k**)

Yellow solid, yield: 67%. ^1^H NMR (600 MHz, CDCl_3_) 0.78-0.88 (m, 7H), 1.00-1.03 (m, 1H), 1.12-1.17 (m, 1H), 1.34-1.37 (m, 2H), 1.40 (s, 3H), 1.43-1.50 (m, 2H), 1.55-1.61 (m, 1H), 1.79-1.82 (m, 1H), 1.96-2.00 (m, 1H), 2.29-2.34 (m, 1H), 2.53-2.56 (m, 1H), 3.59-3.62 (m, 1H), 3.73 (s, 3H), 3.83-3.87 (m, 1H), 4.05-4.09 (m, 1H), 4.15-4.18 (m, 1H), 4.74 (d, *J* = 2.0 Hz, 1H), 5.14 (s, 1H), 5.52 (s, 2H), 6.80 (d, *J* = 8.0 Hz, 1H), 6.90 (dd, *J* = 8.0, 2.0 Hz, 1H), 7.35-7.46 (m, 5H), 7.56 (d, *J* = 2.0 Hz, 1H). ^13^C NMR (150 MHz, CDCl_3_) 163.50, 155.71, 144.20, 137.65, 136.19, 128.61, 128.50, 128.39, 117.32, 116.31, 114.37, 109.65, 104.06, 102.25, 87.75, 80.90, 79.38, 64.64, 55.84, 52.37, 44.18, 39.73, 37.29, 36.36, 34.49, 30.71, 26.12, 24.62, 24.45, 20.24, 12.81. HRMS-ESI: m/z Calcd for C_33_H_40_N_2_O_8_Na [M+Na]^+^: 615.2677; Found: 615.2650.


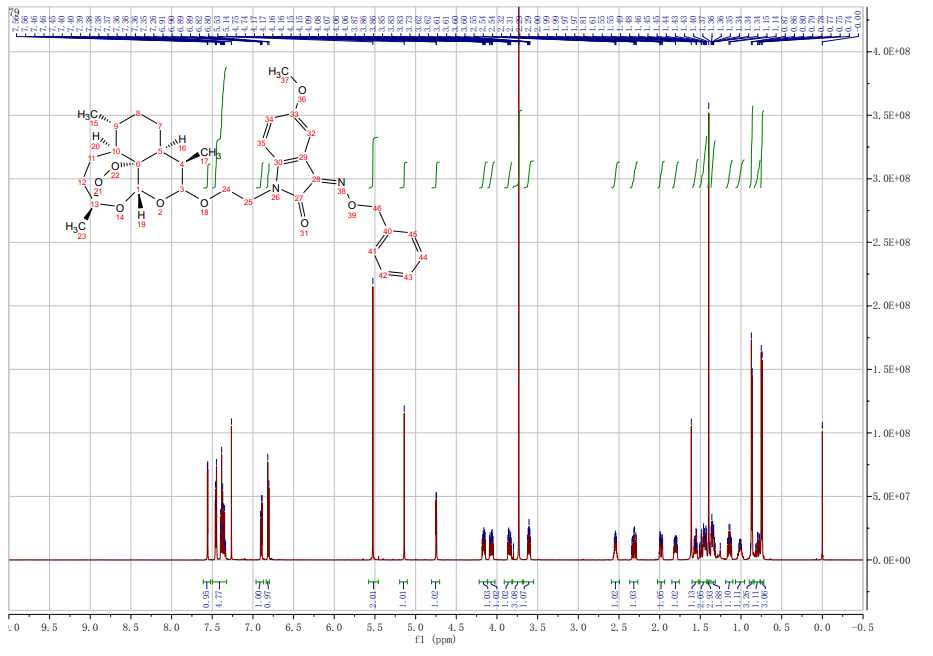


**Figure S31.** The ^1^H NMR spectra of **4k**.


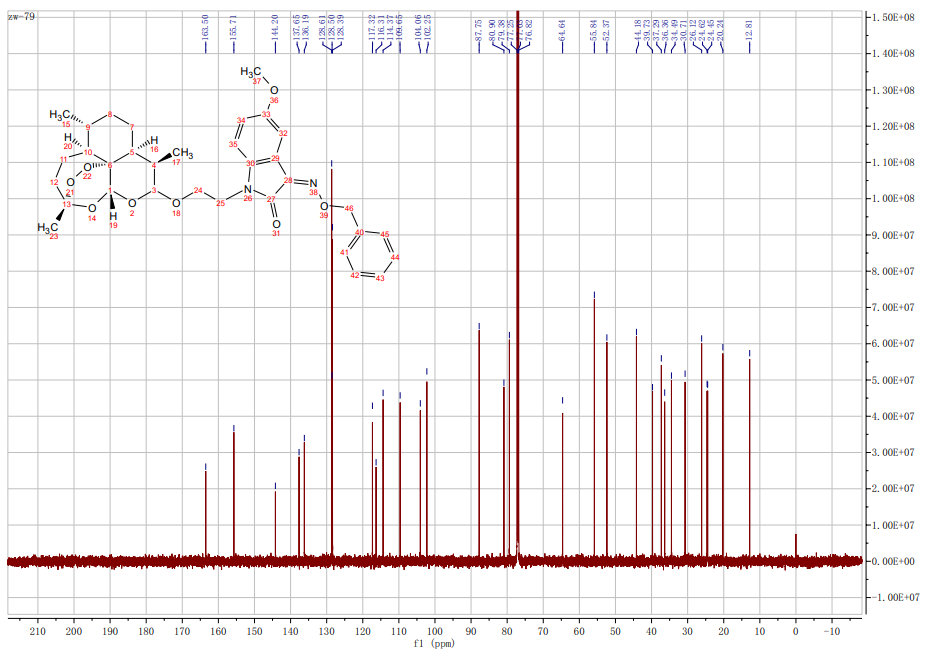


**Figure S32.** The ^13^C NMR spectra of **4k**.


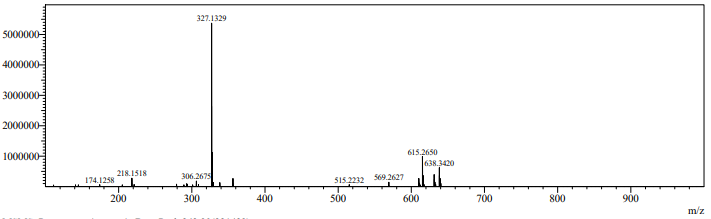


**Figure S33.** The HRMS spectra of **4k**.

**Table S1.** List of potential targets predicted by Target Prediction for 4a

| Target | Common name | Uniprot ID |
| --- | --- | --- |
| Adenosine A2a receptor | *ADORA2A* | P29274 |
| P2X purinoceptor 3 | *P2RX3* | P56373 |
| Caspase-8 | *CASP8* | Q14790 |
| Cathepsin S | *CTSS* | P25774 |
| Insulin-like growth factor I receptor | *IGF1R* | P08069 |
| Dual specificity protein phosphatase 3 | *DUSP3* | P51452 |
| Histone acetyltransferase p300 | *EP300* | Q09472 |
| Induced myeloid leukemia cell differentiation protein Mcl-1 | *MCL1* | Q07820 |
| Calpain 2 | *CAPN2* | P17655 |
| Calpain 1 | *CAPN1* | P07384 |
| Cyclin-dependent kinase 2/cyclin A | *CDK2* | P24941 |
| Cyclin-dependent kinase 2/cyclin A | *CCNA1* | P78396 |
| Cyclin-dependent kinase 2/cyclin A | *CCNA2* | P20248 |
| Tyrosine-protein kinase SYK | *SYK* | P43405 |
| Cyclin-dependent kinase 2 | *CDK2* | P24941 |
| Serine/threonine-protein kinase Aurora-A | *AURKA* | O14965 |
| Cyclin-dependent kinase 5/CDK5 activator 1 | *CDK5* | Q00535 |
| Cyclin-dependent kinase 5/CDK5 activator 1 | *CDK5R1* | Q15078 |
| Gamma-secretase | *PSEN2* | P49810 |
| Gamma-secretase | *PSENEN* | Q9NZ42 |
| Gamma-secretase | *NCSTN* | Q92542 |
| Gamma-secretase | *APH1A* | Q96BI3 |
| Gamma-secretase | *PSEN1* | P49768 |
| Gamma-secretase | *APH1B* | Q8WW43 |
| MAP kinase p38 alpha | *MAPK14* | Q16539 |
| Tyrosine-protein kinase JAK2 | *JAK2* | O60674 |
| Matrix metalloproteinase 2 | *MMP2* | P08253 |
| ADAMTS5 | *ADAMTS5* | Q9UNA0 |
| Alpha-1a adrenergic receptor | *ADRA1A* | P35348 |
| Alpha-1b adrenergic receptor | *ADRA1B* | P35368 |
| Phenylalanyl-tRNA synthetase mitochondrial | *FARS2* | O95363 |
| Arachidonate 12-lipoxygenase | *ALOX12* | P18054 |
| Nicotinamide phosphoribosyltransferase | *NAMPT* | P43490 |
| Tyrosine-protein kinase JAK3 | *JAK3* | P52333 |
| Serine/threonine-protein kinase Aurora-B | *AURKB* | Q96GD4 |
| Nerve growth factor receptor Trk-A | *NTRK1* | P04629 |
| MAP kinase ERK2 | *MAPK1* | P28482 |
| Sodium channel protein type IX alpha subunit | *SCN9A* | Q15858 |
| Signal transducer and activator of transcription 5B | *STAT5B* | P51692 |
| Cyclin-dependent kinase 2/cyclin E | *CCNE2* | O96020 |
| Cyclin-dependent kinase 2/cyclin E | *CDK2* | P24941 |
| Cyclin-dependent kinase 2/cyclin E | *CCNE1* | P24864 |
| Interleukin-8 receptor B | *CXCR2* | P25025 |
| Interleukin-8 receptor A | *CXCR1* | P25024 |
| Liver glycogen phosphorylase | *PYGL* | P06737 |
| Heme oxygenase 1 (by homology) | *HMOX1* | P09601 |
| Cannabinoid receptor 1 | *CNR1* | P21554 |
| Norepinephrine transporter | *SLC6A2* | P23975 |
| Serotonin transporter | *SLC6A4* | P31645 |
| ADAMTS4 | *ADAMTS4* | O75173 |
| Dopamine transporter | *SLC6A3* | Q01959 |
| Matrix metalloproteinase 13 | *MMP13* | P45452 |
| Serine/threonine-protein kinase B-raf | *BRAF* | P15056 |
| Inosine-5'-monophosphate dehydrogenase 2 | *IMPDH2* | P12268 |
| Androgen Receptor | *AR* | P10275 |
| c-Jun N-terminal kinase 1 | *MAPK8* | P45983 |
| c-Jun N-terminal kinase 3 | *MAPK10* | P53779 |
| Matrix metalloproteinase 1 | *MMP1* | P03956 |
| Prolyl endopeptidase | *PREP* | P48147 |
| Bradykinin B1 receptor | *BDKRB1* | P46663 |
| Insulin receptor | *INSR* | P06213 |
| Mineralocorticoid receptor | *NR3C2* | P08235 |
| Glucocorticoid receptor | *NR3C1* | P04150 |
| Progesterone receptor | *PGR* | P06401 |
| Vascular endothelial growth factor receptor 2 | *KDR* | P35968 |
| Fibroblast growth factor receptor 1 | *FGFR1* | P11362 |
| Fructose-1,6-bisphosphatase | *FBP1* | P09467 |
| Type-1 angiotensin II receptor (by homology) | *AGTR1* | P30556 |
| Thromboxane-A synthase | *TBXAS1* | P24557 |
| Adenosine A1 receptor (by homology) | *ADORA1* | P30542 |
| Lysosomal Pro-X carboxypeptidase | *PRCP* | P42785 |
| Telomerase reverse transcriptase | *TERT* | O14746 |
| Matrix metalloproteinase 8 | *MMP8* | P22894 |
| Epidermal growth factor receptor erbB1 | *EGFR* | P00533 |
| Nuclear receptor subfamily 4 group A member 1 | *NR4A1* | P22736 |
| P-glycoprotein 1 | *ABCB1* | P08183 |
| Voltage-gated potassium channel subunit Kv1.3 (by homology) | *KCNA3* | P22001 |
| Protein tyrosine kinase 2 beta | *PTK2B* | Q14289 |
| Matrix metalloproteinase 9 | *MMP9* | P14780 |
| ADAM17 | *ADAM17* | P78536 |
| Cyclin-dependent kinase 2/cyclin E1 | *CDK2* | P24941 |
| Cyclin-dependent kinase 2/cyclin E1 | *CCNE1* | P24864 |
| Tyrosine-protein kinase receptor FLT3 | *FLT3* | P36888 |
| Kinesin-1 heavy chain/ Tyrosine-protein kinase receptor RET | *RET* | P07949 |
| Protein farnesyltransferase | *FNTB* | P49356 |
| Protein farnesyltransferase | *FNTA* | P49354 |
| Tyrosine-protein kinase LCK | *LCK* | P06239 |
| Fibroblast growth factor receptor 3 | *FGFR3* | P22607 |
| Muscle, skeletal receptor tyrosine protein kinase | *MUSK* | O15146 |
| Cyclin-dependent kinase 4/cyclin D1 | *CCND1* | P24385 |
| Cyclin-dependent kinase 4/cyclin D1 | *CDK4* | P11802 |
| Tyrosine-protein kinase YES | *YES1* | P07947 |
| HERG | *KCNH2* | Q12809 |
| Myosin light chain kinase, smooth muscle | *MYLK* | Q15746 |
| Adenosine A2b receptor | *ADORA2B* | P29275 |
| Matrix metalloproteinase 3 | *MMP3* | P08254 |
| Matrix metalloproteinase 7 | *MMP7* | P09237 |
| Toll-like receptor (TLR7/TLR9) | *TLR9* | Q9NR96 |
| Arachidonate 5-lipoxygenase | *ALOX5* | P09917 |
| Serine/threonine-protein kinase mTOR | *MTOR* | P42345 |
| Alpha-ketoglutarate-dependent dioxygenase alkB homolog 3 | *ALKBH3* | Q96Q83 |
| Hepatocyte growth factor receptor | *MET* | P08581 |
| PI3-kinase p110-alpha subunit | *PIK3CA* | P42336 |
| Cathepsin (B and K) | *CTSB* | P07858 |
| Carnitine O-palmitoyltransferase 1, liver isoform | *CPT1A* | P50416 |
| Tyrosine-protein kinase ABL | *ABL1* | P00519 |
| Vascular endothelial growth factor receptor 3 | *FLT4* | P35916 |
| Phosphorylase kinase gamma subunit 2 | *PHKG2* | P15735 |
| Tyrosine-protein kinase SRC | *SRC* | P12931 |
| Focal adhesion kinase 1 | *PTK2* | Q05397 |
| Mitogen-activated protein kinase kinase kinase 9 | *MAP3K9* | P80192 |
| Tyrosine-protein kinase ITK/TSK | *ITK* | Q08881 |
| Interleukin-6 receptor subunit beta | *IL6ST* | P40189 |

**Table S2.** List of cross-over genes of 4a

| Number | Common name |
| --- | --- |
| 1 | *PIK3CA* |
| 2 | *EGFR* |
| 3 | *RET* |
| 4 | *CASP8* |
| 5 | *BRAF* |
| 6 | *AR* |
| 7 | *MET* |
| 8 | *TERT* |
| 9 | *CCND1* |
| 10 | *FGFR3* |
| 11 | *CDK4* |
| 12 | *PGR* |
| 13 | *EP300* |
| 14 | *MTOR* |
| 15 | *AURKA* |
| 16 | *SRC* |
| 17 | *MAPK1* |
| 18 | *FGFR1* |
| 19 | *MMP2* |
| 20 | *ABCB1* |
| 21 | *MMP9* |
| 22 | *IGF1R* |
| 23 | *KDR* |
| 24 | *JAK2* |
| 25 | *MMP1* |
| 26 | *ABL1* |
| 27 | *CDK2* |
| 28 | *PTK2* |
| 29 | *MAPK8* |
| 30 | *FLT4* |
| 31 | *CCNA2* |
| 32 | *CCNE1* |
| 33 | *MAPK14* |
| 34 | *NTRK1* |
| 35 | *MCL1* |
| 36 | *MMP7* |
| 37 | *INSR* |
| 38 | *MMP3* |
| 39 | *CTSB* |
| 40 | *MMP13* |
| 41 | *STAT5B* |
| 42 | *NR3C1* |
| 43 | *MAPK10* |
| 44 | *PTK2B* |
| 45 | *AURKB* |
| 46 | *HMOX1* |
| 47 | *SYK* |
| 48 | *PSEN2* |
| 49 | *NAMPT* |
| 50 | *MYLK* |
| 51 | *ADAM17* |

**Table S3.** Specific node properties of protein-protein interaction network of candidate targets.

| Targets | Degree value | Betweenness centrality | Closeness centrality |
| --- | --- | --- | --- |
| *SRC* | 42 | 215.4892 | 0.875 |
| *EGFR* | 41 | 195.4117 | 0.859649 |
| *CCND1* | 40 | 217.5689 | 0.844828 |
| *MTOR* | 36 | 96.03968 | 0.790323 |
| *MAPK1* | 34 | 100.9017 | 0.765625 |
| *PIK3CA* | 33 | 76.19692 | 0.753846 |
| *MMP9* | 30 | 100.869 | 0.720588 |
| *MAPK8* | 26 | 40.18021 | 0.680556 |
| *MMP2* | 25 | 46.66616 | 0.671233 |
| *MAPK14* | 25 | 73.09531 | 0.671233 |
| *JAK2* | 24 | 25.25114 | 0.662162 |
| *AR* | 24 | 16.92872 | 0.662162 |
| *MCL1* | 24 | 29.27302 | 0.662162 |
| *CASP8* | 23 | 27.50516 | 0.653333 |
| *STAT5B* | 23 | 27.6856 | 0.644737 |
| *IGF1R* | 22 | 11.69442 | 0.644737 |
| *CCNE1* | 22 | 20.57007 | 0.644737 |
| *KDR* | 22 | 28.2543 | 0.644737 |
| *EP300* | 22 | 14.82028 | 0.644737 |
| *PGR* | 21 | 18.91774 | 0.636364 |
| *MET* | 21 | 16.56831 | 0.636364 |
| *CDK4* | 21 | 12.79909 | 0.636364 |
| *PTK2* | 20 | 24.84961 | 0.628205 |
| *ABL1* | 20 | 10.55221 | 0.628205 |
| *CCNA2* | 19 | 9.256352 | 0.620253 |
| *CDK2* | 19 | 10.57459 | 0.620253 |
| *TERT* | 18 | 10.86779 | 0.604938 |
| *FGFR1* | 17 | 8.683085 | 0.597561 |
| *NTRK1* | 15 | 7.372299 | 0.590361 |
| *RET* | 14 | 3.113384 | 0.576471 |
| *MMP1* | 14 | 18.58879 | 0.583333 |
| *NR3C1* | 13 | 4.582048 | 0.576471 |
| *PTK2B* | 11 | 3.34678 | 0.550562 |
| *FLT4* | 11 | 1.604977 | 0.556818 |
| *FGFR3* | 11 | 2.426775 | 0.556818 |
| *AURKA* | 11 | 2.332488 | 0.556818 |
| *MMP3* | 11 | 5.248165 | 0.563218 |
| *SYK* | 10 | 1.507703 | 0.550562 |
| *HMOX1* | 10 | 1.827778 | 0.556818 |
| *MMP7* | 10 | 3.08814 | 0.556818 |
| *BRAF* | 9 | 0.633333 | 0.544444 |
| *AURKB* | 9 | 7.80404 | 0.526882 |
| *ADAM17* | 9 | 13.8235 | 0.544444 |
| *ABCB1* | 9 | 1.159199 | 0.544444 |
| *INSR* | 8 | 0.086957 | 0.532609 |
| *CTSB* | 8 | 5.205386 | 0.521277 |
| *MAPK10* | 7 | 0.317647 | 0.526882 |
| *MMP13* | 4 | 0.793651 | 0.462264 |
| *MYLK* | 4 | 1.383808 | 0.505155 |
| *PSEN2* | 2 | 0.282828 | 0.471154 |

**Table S4.** The MM-PBSA analysis of **4a**-key target protein

| Energy | **4a**-EGFR | **4a**-MAPK8 | **4a**-PIK3CA |
| --- | --- | --- | --- |
| Van der Waals Energy (kJ/mol) | -223.782 | -194.114 | -194.533 |
| Electrostatic energy (kJ/mol) | -31.230 | -10.179 | -31.394 |
| Polar solvation energy (kJ/mol) | 185.099 | 101.993 | 125.980 |
| Nonpolar solvation Energy (kJ/mol) | -24.451 | -23.245 | -25.574 |
| Total Binding Energy (kJ/mol) | -94.364 | -125.544 | -125.521 |
| T∆S (kJ/mol) | 22.054 | 15.750 | 14.688 |
| Total Binding Free Energy (kJ/mol) | -72.310 | -109.794 | -110.833 |


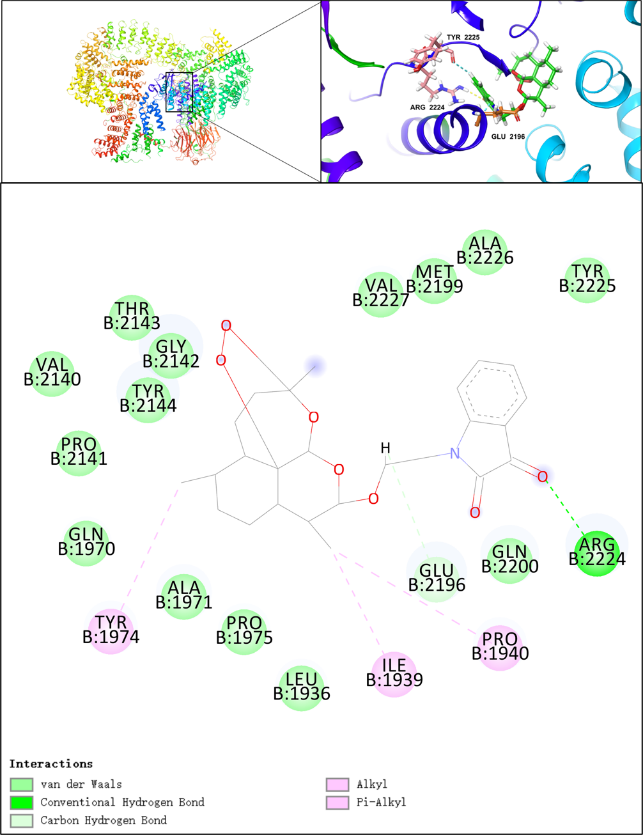


**Figure S34.** Image of the docking results of 4a-MTOR.


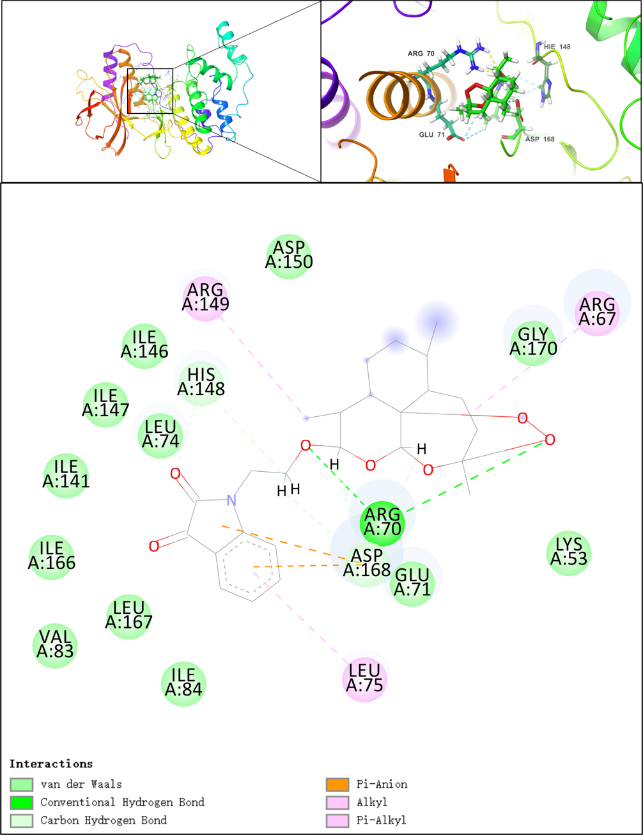


**Figure S35.** Image of the docking results of 4a-MAPK14.


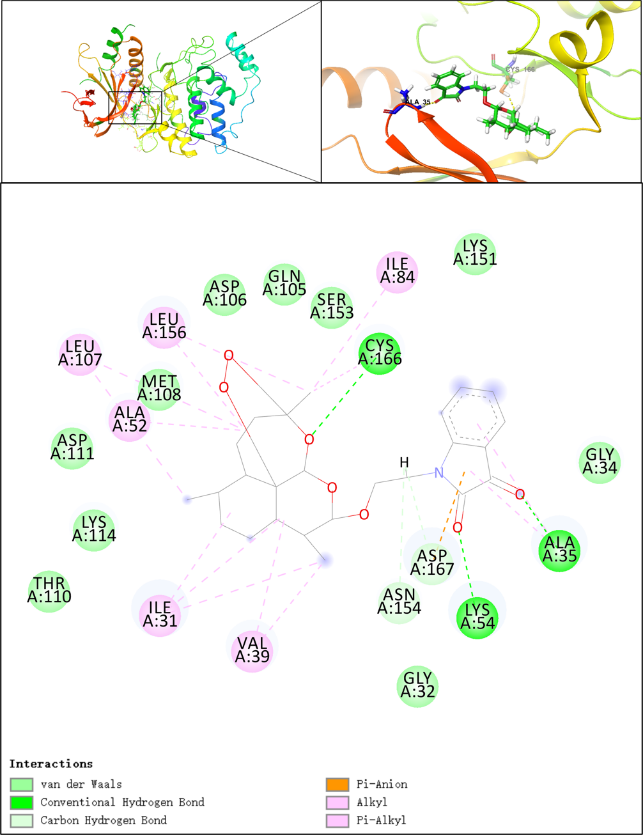


**Figure S36.** Image of the docking results of 4a-MAPK1.


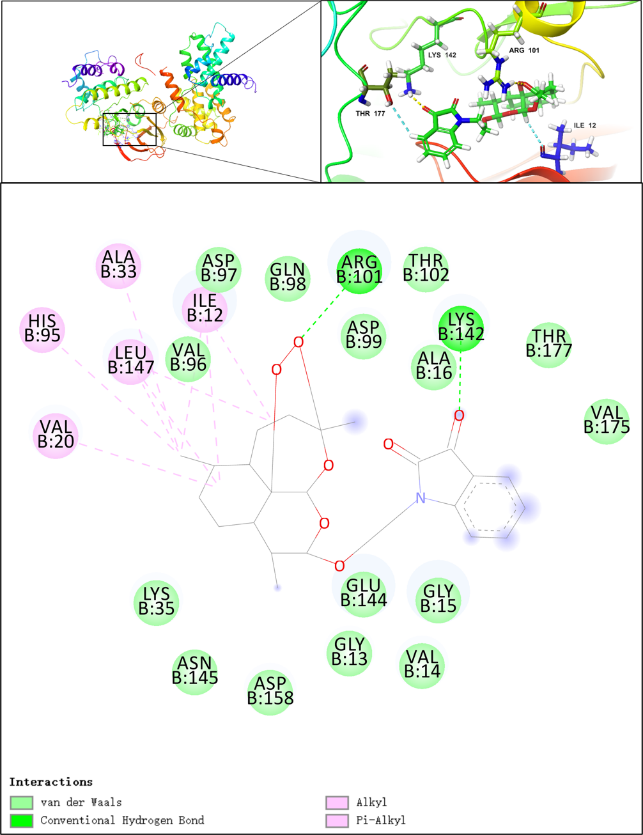


**Figure S37.** Image of the docking results of 4a-CCND1.


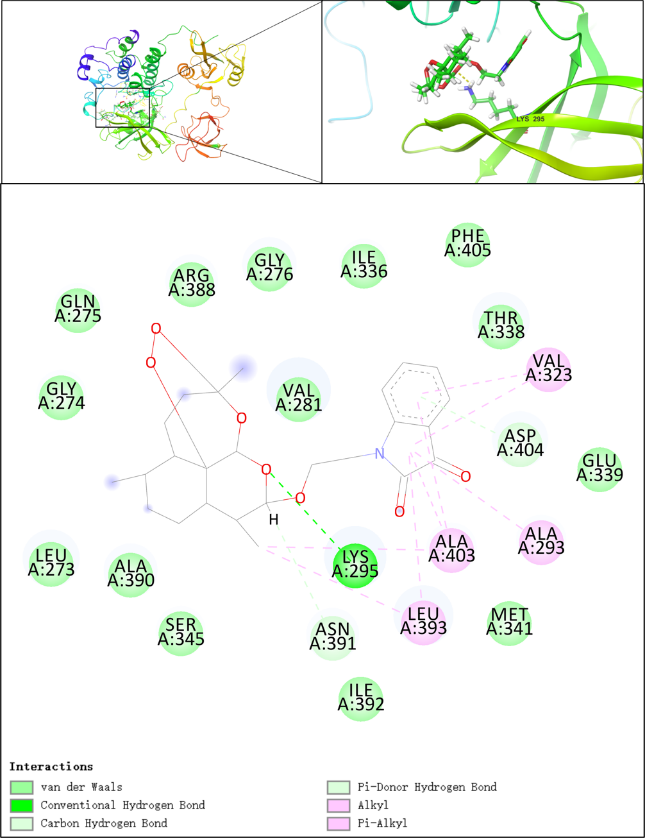


**Figure S38.** Image of the docking results of 4a-SRC.


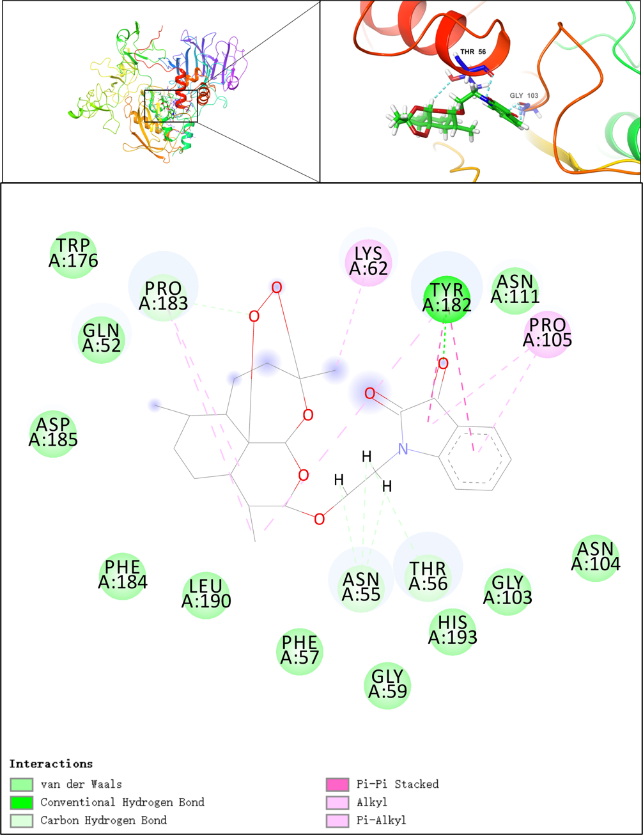


**Figure S39.** Image of the docking results of 4a-MMP2.


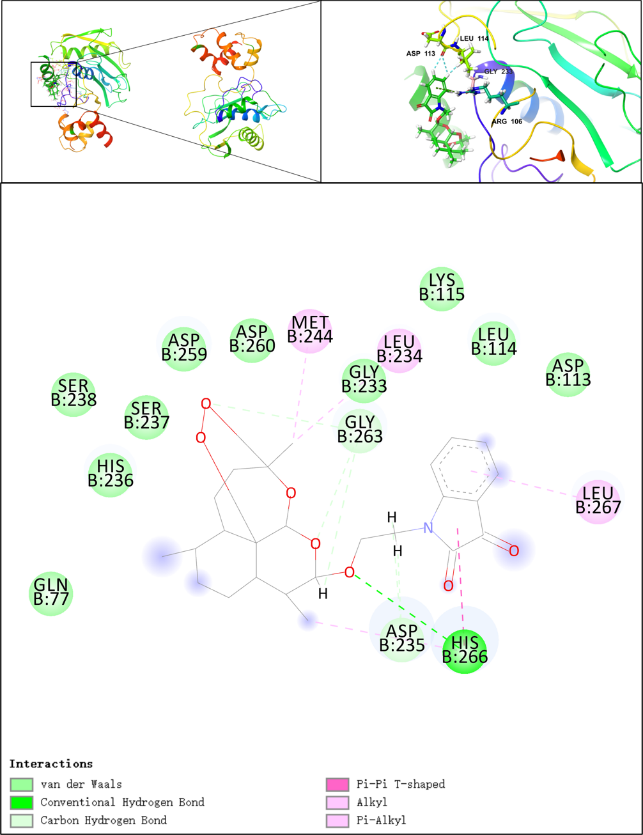


**Figure S40.** Image of the docking results of 4a-MMP9.


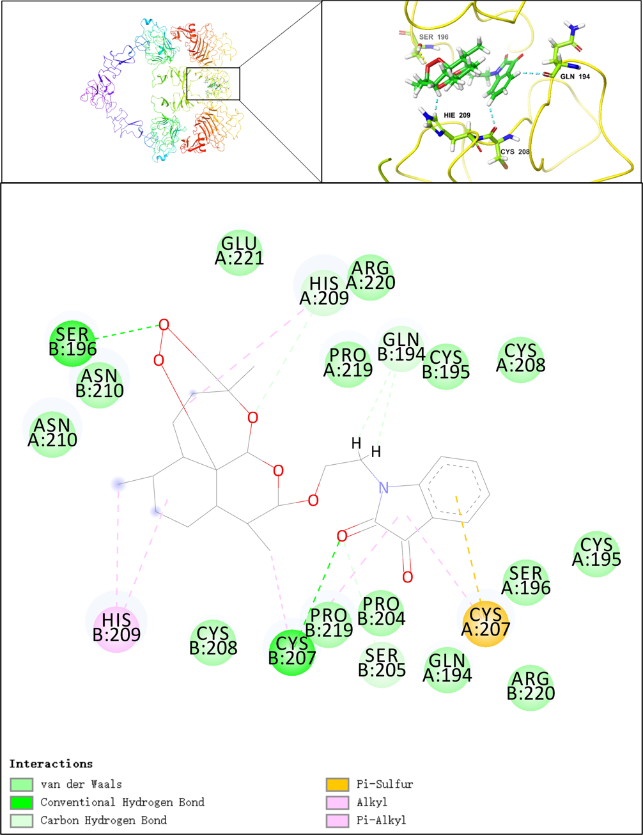


**Figure S41.** Image of the docking results of 4a-EGFR.


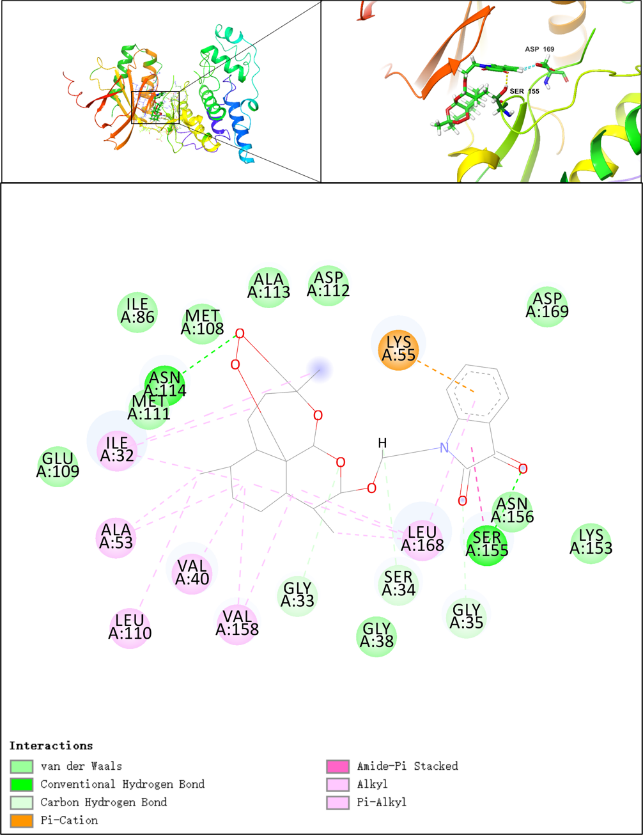


**Figure S42.** Image of the docking results of 4a-MAPK8.


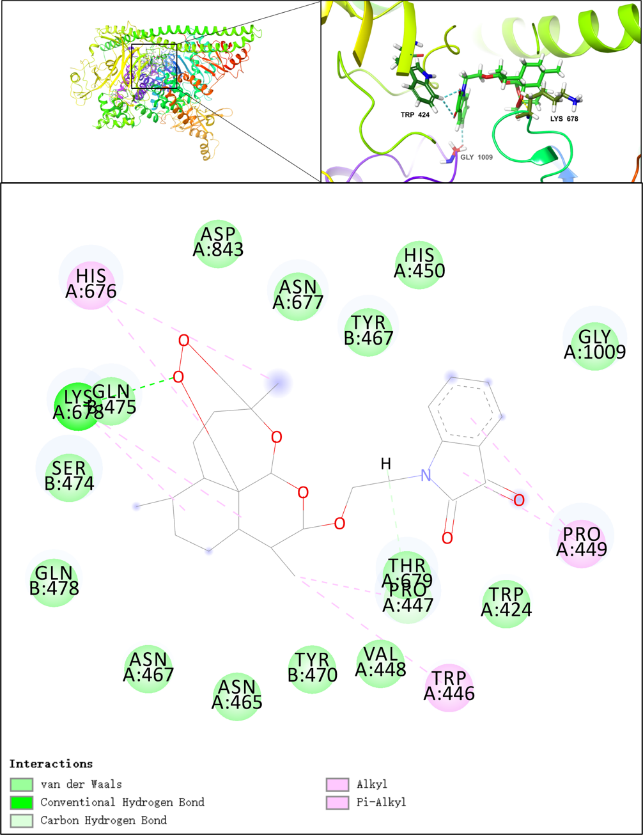


**Figure S43.** Image of the docking results of 4a-PIK3CA.


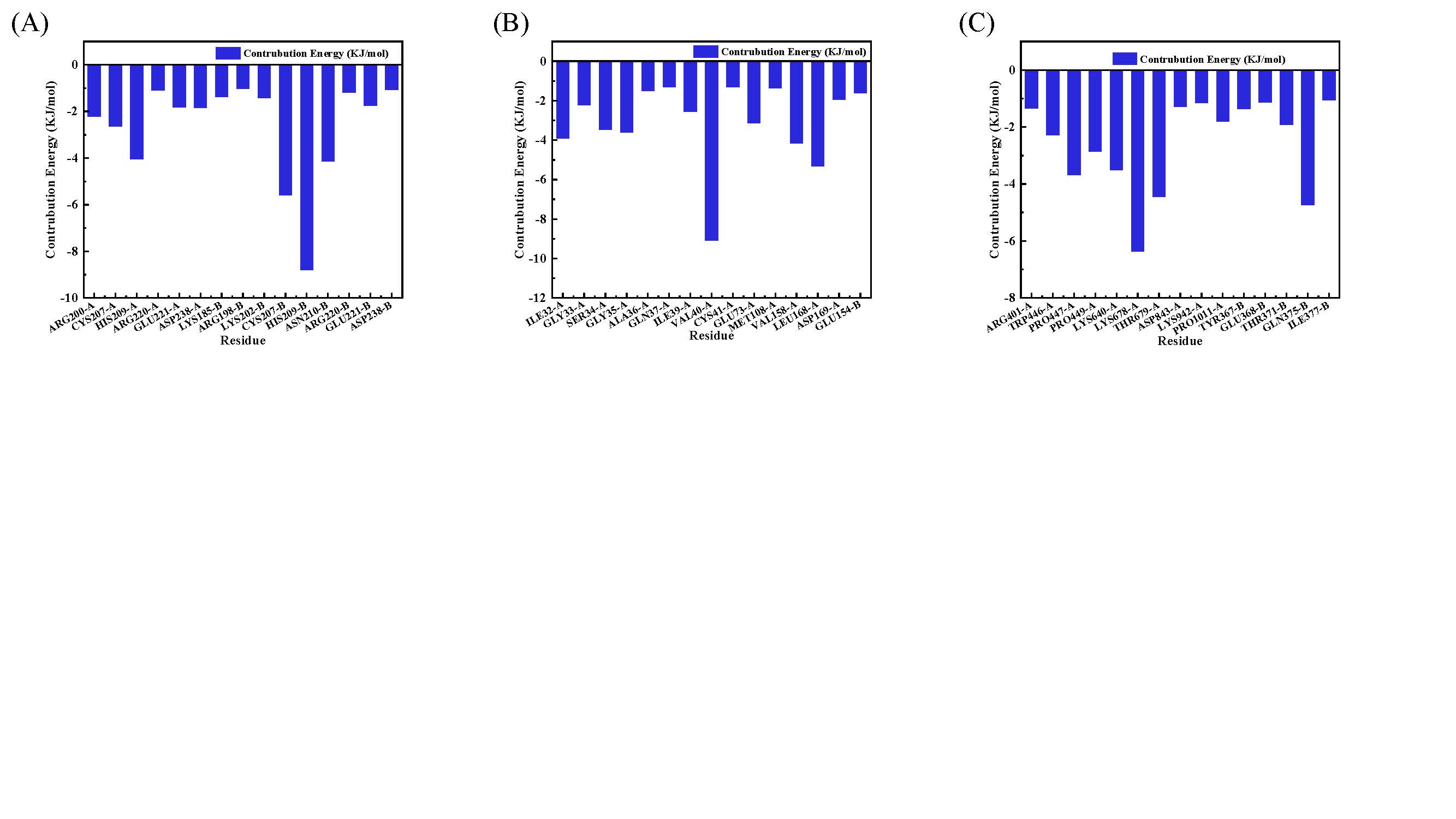


**Figure S44.** Binding energy distribution of the main residues of the (A) 4a-EGFR complex during simulation process. (B) 4a-MAPK8 complex during simulation process. (C) 4a-PIK3CA complex during simulation process.

Reference

1. Wang, R.; Zhang, Q.; Chen, M., Artemisinin‐isatin hybrids tethered via ethylene linker and their anti‐lung cancer activity. *Archiv der Pharmazie* **2023,** *356* (4), 2200563.

2. Daina, A.; Michielin, O.; Zoete, V., SwissTargetPrediction: updated data and new features for efficient prediction of protein targets of small molecules. *Nucleic acids research* **2019,** *47* (W1), W357-W364.

3. Oliveros, J. C., VENNY. An interactive tool for comparing lists with Venn Diagrams. *http;//bioinfogp.cnb.csic.es/tools/venny/index. html* **2007**.

4. Szklarczyk, D.; Gable, A. L.; Lyon, D.; Junge, A.; Wyder, S.; Huerta-Cepas, J.; Simonovic, M.; Doncheva, N. T.; Morris, J. H.; Bork, P., STRING v11: protein–protein association networks with increased coverage, supporting functional discovery in genome-wide experimental datasets. *Nucleic acids research* **2019,** *47* (D1), D607-D613.

5. Sherman, B. T.; Hao, M.; Qiu, J.; Jiao, X.; Baseler, M. W.; Lane, H. C.; Imamichi, T.; Chang, W., DAVID: a web server for functional enrichment analysis and functional annotation of gene lists (2021 update). *Nucleic acids research* **2022,** *50* (W1), W216-W221.

6. Valdés-Tresanco, M. S.; Valdés-Tresanco, M. E.; Valiente, P. A.; Moreno, E., gmx_MMPBSA: a new tool to perform end-state free energy calculations with GROMACS. *Journal of chemical theory and computation* **2021,** *17* (10), 6281-6291.
